# Differential Repair Protein Recruitment at Sites of Clustered and Isolated DNA Double-Strand Breaks Produced by High-Energy Heavy Ions

**DOI:** 10.1038/s41598-020-58084-6

**Published:** 2020-01-29

**Authors:** Burkhard Jakob, Monika Dubiak-Szepietowska, Ellen Janiel, Alina Schmidt, Marco Durante, Gisela Taucher-Scholz

**Affiliations:** 1 Department of Biophysics, GSI Helmholtzzentrum für Schwerionenforschung, 64291 Darmstadt, Germany; 2 Department of Biology, Technische Universität Darmstadt, 64287 Darmstadt, Germany; 3 Department of Physics, Technische Universität Darmstadt, 64287 Darmstadt, Germany

**Keywords:** Biophysics, Cancer, Cell biology

## Abstract

DNA double-strand break (DSB) repair is crucial to maintain genomic stability. The fidelity of the repair depends on the complexity of the lesion, with clustered DSBs being more difficult to repair than isolated breaks. Using live cell imaging of heavy ion tracks produced at a high-energy particle accelerator we visualised simultaneously the recruitment of different proteins at individual sites of complex and simple DSBs in human cells. NBS1 and 53BP1 were recruited in a few seconds to complex DSBs, but in 40% of the isolated DSBs the recruitment was delayed approximately 5 min. Using base excision repair (BER) inhibitors we demonstrate that some simple DSBs are generated by enzymatic processing of base damage, while BER did not affect the complex DSBs. The results show that DSB processing and repair kinetics are dependent on the complexity of the breaks and can be different even for the same clastogenic agent.

## Introduction

Repair of DNA double-strand breaks is a necessary pathway to maintain genomic stability in mammalian cells^1^. The DNA damage response (DDR) signalling cascade is rapid and hierarchically coordinated, with many proteins being recruited to the damage sites at different times and depending on the repair pathway. Complex DSBs, i.e. complex lesions involving multiple strand breaks and/or oxidative damages within two helical turns^2,3^, are more difficult to repair than isolated, frank DSBs^4^. Complex DSBs can be produced by ionising radiation (IR)^3,5^, and their fraction and complexity and clustering increases for densely ionising, high-linear energy transfer (LET) radiation such as α-particles and heavy ions^6^. Endogenous DSBs (simple DSBs produced during replication) have much higher frequency than IR-induced DSBs at low doses, but the latter include complex DSBs^7^. These complex lesions are considered ultimately responsible for the late effects of low doses of IR^8^, including environmental exposures and cosmic radiation risk in space travel. The repair of simple and complex DSBs is therefore crucial to understand the difference between endogenous and exogenous genotoxicity and for modelling the risk related to exposure to low doses IR on Earth^9^ and in space^10^.

Delayed repair of clustered DSBs has been observed by immunostaining of markers of single strand breaks (SSBs), DSBs and base damage in mammalian cells^11^. Here we measured the early protein recruitment at sites of simple and clustered, complex DSBs by live cell imaging. Immunostaining on fixed samples is unable to identify the early kinetics and cannot follow the evolution of individual foci, therefore providing only average values^12^. High-charge and -energy (HZE) ions offer a unique opportunity for these studies as they simultaneously produce both clustered (along the track) and isolated (off-track) DSBs. In fact, the inner part of the ion track (core) includes primary particle ionizations and low-energy electrons, and results mostly in complex, clustered DSBs for HZE ions at high linear energy transfer (LET), whereas low-LET high-energy ionised electrons (δ-ray) around the primary track (penumbra) produce mostly simple DSBs and only few complex, non-clustered DSBs at the end of their range, similar to the damage produced by X-rays (Supplemental Fig. S1). The overlap of δ-rays produced by different tracks increase the frequency of off-track DSBs that can be recorded (Supplemental Fig. S2). Live cell imaging of protein recruited to the track core has been used by us and others to measure the movement of the DSB lesions in the cell nucleus^13–15^. We have previously shown that the yield of heavy ion-induced DSBs as a function of the distance from the primary track can be well described by the physics of the energy deposition of the primary ion and δ-rays^16^. To measure the repair kinetics in single DSB sites we have now exposed to swift heavy ions human osteosarcoma (U2OS) cells stably expressing the GFP-tagged DSB repair factors NBS^17^ and 53BP1^18^, two different DSB surrogate markers which were recruited in all cell cycle phases. Individual foci in core and penumbra of the track were followed by live cell imaging from a few seconds up to 45 min post-irradiation. In this time frame we could visualise the early recruitment of repair factors and the release of the proteins after completion of repair in individual isolated and clustered DSBs. A similar setup was implemented for irradiation with X-rays using the same cells for comparison.

## Results

### Differences in lesion complexity within individual HZE particle tracks impact on the timing of the DDR

**Figure 1:**
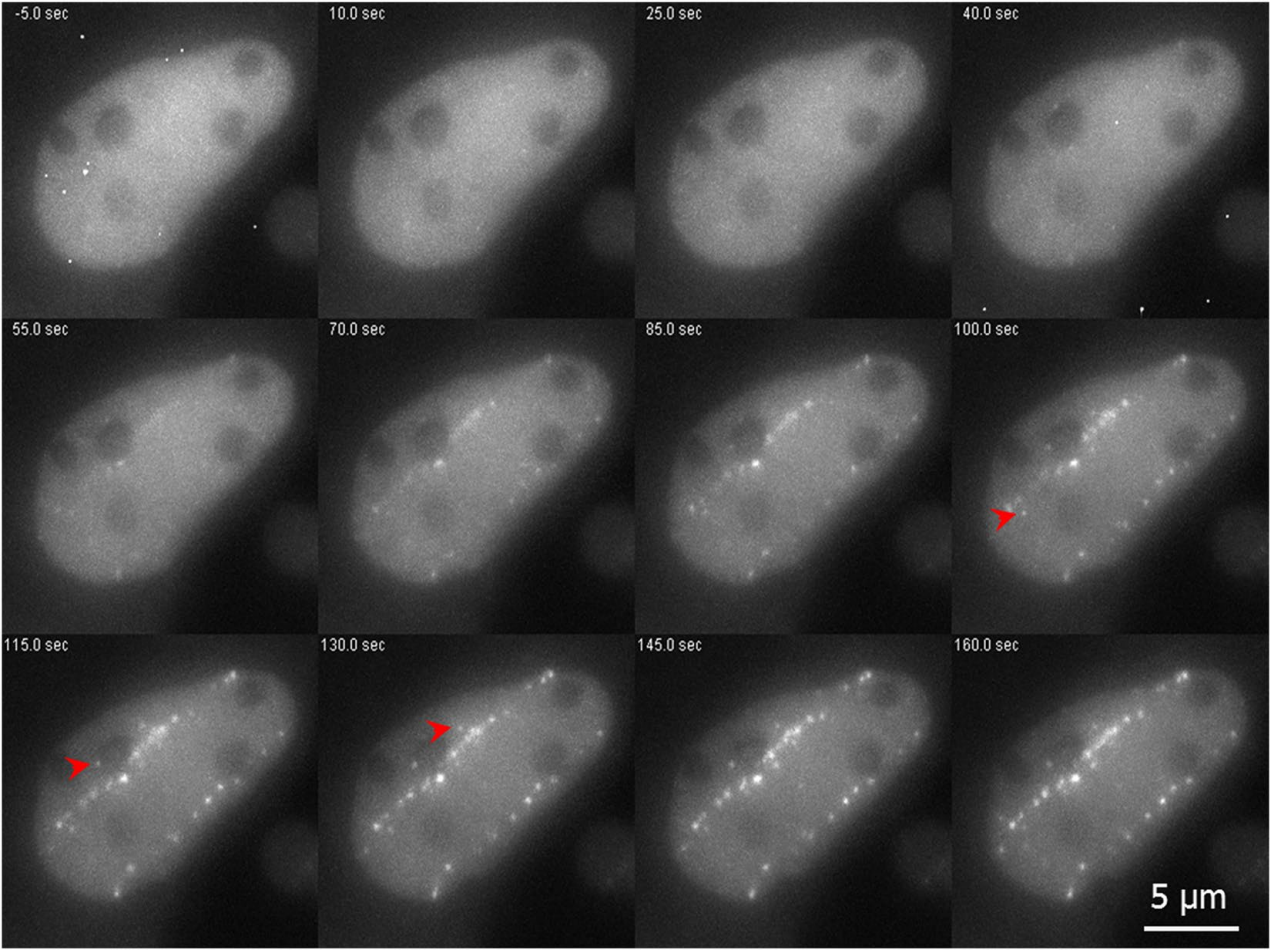
DSB repair factor NBS1-GFP is recruited to HZE ion tracks. Live cell imaging of GFP tagged NBS1 in U2OS cells revealed fast foci formation along the trajectories of ions traversing the nucleus after irradiation with 1 GeV/u Fe ions. The slightly delayed development of some clearly off-track foci (arrowheads) could be observed. Selected time-frames are shown. Image montage was done using ImageJ 1.48 v (https://imagej.nih.gov) and Powerpoint 2010 (Microsoft Corporation, USA)- for full movie see Supplement Movie 1.

To investigate the influence of simple and complex DNA lesions on the early DNA damage response (DDR) we made use of the different parts of the radiation tracks of HZE ions delivered by a particle accelerator in combination with real time live cell microscopy. For our study we used the established human osteosarcoma (U2OS) cell lines stably expressing the GFP-tagged DSB repair factors NBS1 and 53BP1 to follow the recruitment of these factors to the damage sites after irradiation. After irradiation with 1 GeV/u Fe-ions, we observed a fast recruitment of NBS1-GFP to the charged particle induced dense damage along the ion track. Protein accumulation at the track becomes clearly visible around 50 s after the ion traversal (Fig. 1; for full time series see Supplemental Movie 1).

**Figure 2:**
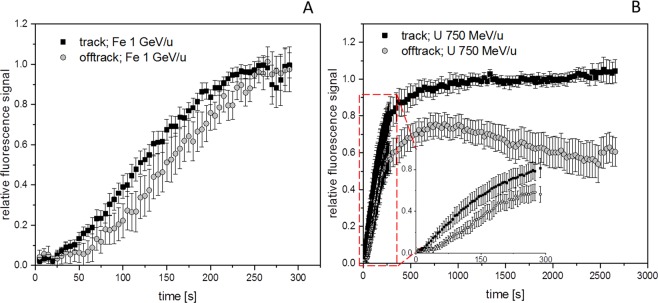
Kinetics of NBS1 binding to HZE induced DNA damage. Quantitative analysis of nuclear wide recruitment kinetics of NBS1-GFP in U2OS cell nuclei. (**A**) At 1 GeV/u Fe trajectories a somehow delayed recruitment for the early observable off-track foci compared to the recruitment associated to the internal part of the track becomes obvious (n = 46 or 88 RIF in 8 nuclei each). A significant deviation of the recruitment curves starts at around 50 s post-irradiation (t-test, p = 0.05). Error bars 95% CI. (**B**) By using 750 MeV/u uranium many off-track foci are generated due to the higher LET, thus facilitating analysis. Recruitment kinetics with delayed off tack foci similar to the ones observed after 1 GeV/u Fe are obtained by nuclear-wide fluorescence analysis at 750 MeV/u U. In addition, longer observation times revealed a transient binding behaviour for the off-track foci compared to a more stable saturation of the fluorescence signal at the ion trajectories (n = 20 nuclei). The deviation of the recruitment curves is significant for t > 20 s (2 sided t-test, p = 0.05). Error bars 95% CI. Graphs were generated using Origin Pro V.2019 (Originlab Corp., Northampton, MA, USA).

Interestingly, some isolated foci (Fig. 1 arrow heads) occurring off-track showing a slightly delayed recruitment of NBS1 to DSBs could be monitored. These sparsely occurring off-track foci are likely to be generated by δ-electron track ends and represent isolated DSBs. For NBS1-GFP, the occurrence of radiation induced foci (RIF) in mock treated samples was a rare event under the applied imaging conditions with most cells showing no foci formation. This indicates that the analysed delayed occurring foci are induced by the application of IR and not light-induced during microscopy (Supplemental Fig. S3). Quantitative analysis of NBS1 intensities at both nuclear wide in-track and off-track DSBs yielded average recruitment curves (Fig. 2A) similar to the ones described in literature for low-energy ions^19^. Whereas the slope of the recruitment is nearly identical for the averaged curves, a slight, but significant delay is evident for the off-track compared to the in-track foci (Fig. 2A) resembling the optical observation. Fitting the recruitment curves to a delayed starting mono-exponential saturation curve yielded nearly identical time constants (96 ± 4 s vs. 97 ± 5 s) but a significant, around 50% longer delay time (61 ± 2 s vs 90 ± 3 s) (Supplemental Fig. S4) Theses results suggest a generally altered timing of the DNA damage response in dependency of lesion complexity.

### Simple off-track DSBs are repaired faster compared to clustered in-track DSBs

**Figure 3:**
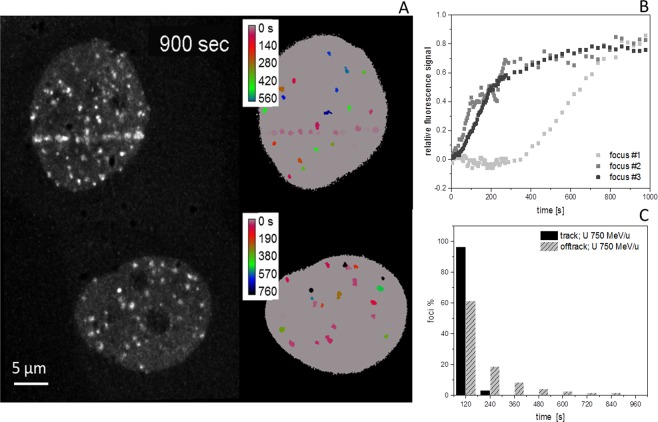
Analysis of individual NBS1 RIF formation kinetics. (**A**) NBS1-GFP fluorescence signal of a U2OS nucleus traversed by a single uranium ion at 750 MeV/u 15 min after irradiation (upper left) and nearby non-traversed nucleus showing off-track foci only (lower left). right: colour coded lag-phases (delay times) for individual selected foci in-track and off-track revealing fast and concerted occurrence of NBS1 recruitment inside track, but a fraction of quite delayed responding off-track RIF with variable delay times. No indication for a radial dependency of lag-phases for the off-track foci or differences in traversed vs. non traversed nuclei could be observed. (**B**) Fluorescence intensity traces for the recruitment of NBS1 to selected individual off-track DSBs showing clear indication of delayed responding foci. (**C**) Distribution of lag phases for in- track and off-track foci (n = 88 or 189, respectively) showing a fraction of around 40% of off-track foci responding delayed. The difference of the distributions is highly significant (Mann-Whitney U-test). Image montage and analysis of (**A**) was done using ImageJ 1.48 v (https://imagej.nih.gov). Graphs (**B**,**C**) were generated using Origin Pro V.2019 (Originlab Corp., Northampton, MA, USA).

In order to increase the yield of off-track foci, in the following experiment high-energy uranium ions were used (750 MeV/u; Figs. 2B and 3). In addition, to examine if the different radiation quality in the densely ionising part of the track and off-track effects the persistence of the NBS1 signals at the DNA break sites, we prolonged the post-irradiation observation time. Due to the much higher LET of the U-ions (2020 keV/µm in water) in comparison to the Fe-ions (150 keV/µm), not only more dense and complex damage along the track is generated, but also the deposited off-track dose and thus the probability for the formation of off-track DSBs can be largely increased without increasing the number of direct traversals (Supplemental Fig. S1). After irradiation with uranium, multiple off-track RIF become clearly evident in the nuclei (Fig. 3A; for full time series see Supplemental Movie 1), thus facilitating further quantitative analysis. A high number of radiation- induced NBS1-foci were observed in nuclei not directly traversed by a uranium ion (Fig. 3A, lower nucleus). The cells irradiated with uranium ions reveal again a significantly delayed recruitment of NBS1-GFP to early appearing off-track foci compared to the track core in the nuclear wide analysis (Fig. 2B), similar to the curves obtained after irradiation with 1 GeV/u Fe (Fig. 2A). The off-track foci show a more transient recruitment indicating less persisting NBS1 binding, whereas along the track the signal saturated and remained stable over the whole observation time of around 45 min (Fig. 2B). The lack of persistence of the off-track NBS1-GFP recruitment at early responding foci was confirmed using 350 MeV/u Ni (Supplemental Fig. S5 and Movie 2), indicating that the ion charge and LET do not have a major impact on the observed kinetic differences between core and penumbra DNA damage.

### Individual DSBs in single particle-irradiated nuclei reveal variable delays in repair factor recruitment

**Figure 4:**
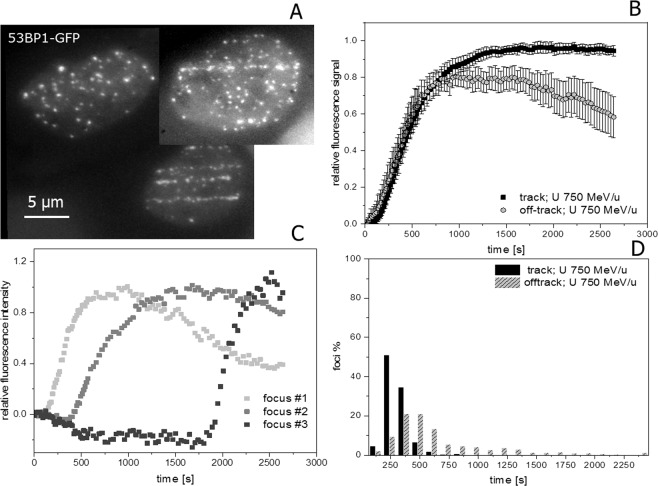
Real time recruitment of 53BP1-GFP in U2OS cells after irradiation with 750 MeV/u uranium ions shows also individually delayed off-track foci. (**A**) U2OS nuclei expressing 53BP1-GFP showing accumulation at DSBs both at ion trajectories as well as off-track. (**B**) Nuclear wide recruitment kinetics of 53BP1 to early occurring DNA DSBs induced by 750 MeV/u U ions is characterized by a more sigmoidal recruitment behavior with a slower accumulation compared to NBS1 (see Fig. 2). Also for 53BP1, the protein accumulation at off-track DSBs appears more transiently (significant for t > 20 min, two-sided t-test p = 0.05) compared to the accumulation at the track with a maximum around 15–20 min post-irradiation, most probably indicating faster repair due to lower complexity. (n = 28 nuclei). Error bars 95% CI. (**C**) Selected traces of 53BP1-GFP recruitment to individual off-track foci showing clearly delayed onset of responses for some off-track DSBs. To better compare the kinetic behaviour, the clearly delayed recruiting DSBs were omitted in the global analysis of B. (**D**) Analysis of lag phases of off-track 53BP1 foci formation showed a broad distribution with a median at 465 s post-irradiation in comparison to 240 s for in track foci. (n = 674 or 274 RIF, respectively). The difference of the distributions is highly significant (Mann-Whitney U-test). Image montage of (**A**) was done using ImageJ 1.48 v (https://imagej.nih.gov). Graphs were generated using Origin Pro V.2019 (Originlab Corp., Northampton, MA, USA).

Whereas hitherto the general description of the dynamic behaviour was based on global (nuclear wide) analysis of NBS1-GFP recruitment to radiation-induced damage sites, in the following we made use of the possibility to track individual foci in the live cell measurements. This allowed us to distinguish and address the recruitment of the damage sensing factor NBS1 to individual damage sites representing single DSBs for the off -track signal. Along the track core, which includes multiple (complex) DSBs in a single RIF or repair centre after high LET particle irradiation according to previous work from our group^20,21^ and others^14,22^, a quite uniform fast and concerted recruitment to individual foci was observed. In contrast, individual off-track foci are characterised by an asynchronous response which is mainly governed by a distribution of individual delays (lag-phases; Fig. 3). Whereas less than 4% of NBS1-RIF inside the track showed a pronounced delay, around 40% of the observed off-track foci displayed lag phases larger than 2 min. The rising slope did not depend on the observed delay times for individual foci and no dependency of the delay to the radial distance from the track was observed for the off-track foci. Furthermore, around 50% of the off-track foci showed a clearly transient binding behaviour compared to <20% inside the track, reflecting the different lesion density and complexity. To rule out that the individually delayed damage response is not a unique feature of NBS1 we irradiated U2OS-cells expressing 53BP1, a mediator and important regulator in the DSB damage response known to form RIF in chromatin surrounding DSBs. After irradiation with 750 MeV/u uranium ions, 53BP1 accumulations along the tracks of traversing particles as well as multiple off-track foci in traversed and non-traversed nuclei could be detected (Fig. 4A for full time series see Supplemental Movie 3).

Besides a slower recruitment (consistent with 53BP1 downstream role in the damage processing) and more pronounced delays, the DSB repair factor 53BP1 showed a similar behaviour as NBS1 regarding a more transient binding at off-track foci (with a maximum around 15–20 min post-irradiation) as well as the occurrence of individually delayed formation of off-track RIF (Fig. 4B,C). Again, quantitative analysis of lag-phases indicates a significant and clear difference between the relatively prompt recruitment of 53BP1 to the complex lesions inside the track and the broad distribution of delays for the off-track foci (Fig. 4D). For 53BP1, around 80% of off-track RIF show delay times >300 s after irradiation with uranium ions compared to only 22% in the track-core having lag phases larger than 300 s. More than 30% off track foci showed a lag phase exceeding 10 min. The 53BP1 kinetics emphasises the variability in the response to individual damage sites thus confirming the general observations obtained with NBS1.

### X-ray induced DSBs are similar to simple off-track DSBs

**Figure 5:**
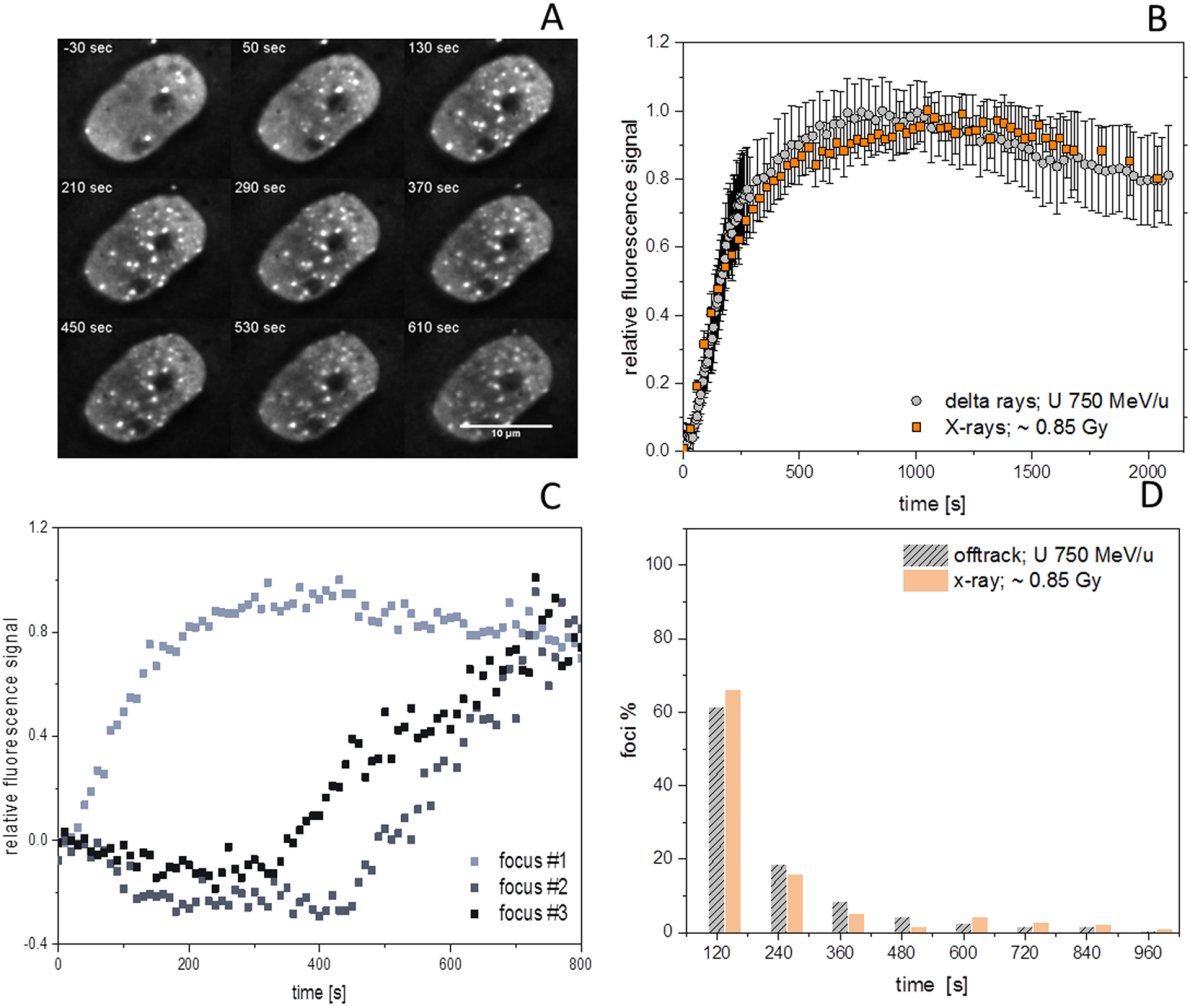
Live cell imaging of real time NBS1 focus formation using X-rays. (**A**) Selected images of a movie showing NBS1-GFP foci formation during radiation with sparsely ionising radiation. (**B**) Nuclear wide analysis of recruitment kinetics showing nearly identical NBS1 recruitment kinetics for X-ray induced (n = 25 up to 10 min and 4–14 for the longer time points) and delta-ray induced DSBs (n = 73; Error bars 95% CI). Data were normalised to their maximum at around 20 min. (**C**) Analysis of NBS1 recruitment kinetics to individual X-ray induced DSBs showing a fraction of clearly delayed recruiting foci. (**D**) Distribution of lag-phases showing similarity after X-rays (n = 333) to the lag phase distribution of off-track- foci after HZE particle irradiation (from Fig. 2C; n = 189). Image montage of (A) was done using ImageJ 1.48 v (https://imagej.nih.gov). Graphs were generated using Origin Pro V.2019 (Originlab Corp., Northampton, MA, USA).

To corroborate if off-track foci represent less complex DNA lesions (isolated DSBs) like the ones induced by low-LET photon irradiation and to confirm the variability of the early DDR response at individual DSBs, we compared the live cell recruitment behaviour of NBS1 evaluated using the same microscope setup, but now coupled to a 35 kV X-ray source delivering relatively high dose rates (around 0.85 Gy/s). During X-ray irradiation we measured the recruitment of NBS1-GFP and 53BP1-GFP to radiation-induced RIF. Mean recruitment kinetics after X-rays match well to the ones observed for off-track foci using charged particles (Fig. 5).

Thus also photon-induced DSBs show a transient binding of NBS1, with a signal intensity peaking at 10–15 min. Moreover, the analysis of single X-ray induced foci revealed a broad range of individually different kinetic traces (Fig. 5C) with some clearly delayed recruiting DSBs. A fraction of ~35% of NBS1 foci were delayed by more than 2 min (Fig. 5D). Compared to NBS1-GFP, 53BP1-GFP showed larger, more clear and distinct RIF after X-ray irradiation, thus facilitating the evaluation (Supplemental Fig. S6A and Movie 4). Whereas mock irradiated samples develop around 1–2 foci/hour on average for 53BP1 under the applied imaging conditions, 0.85 Gy of X-rays induced around 28 foci in accordance with the expected number of DSBs^23,24^ (Supplemental Fig. S3 and S6). Similarly to NBS1, the mean X-ray response of 53BP1 resembles the one obtained for off-track foci produced by HZE ions, supporting the similarity between X- and δ-rays (Supplemental Fig. S6B). In addition, 53BP1 recruitment to individual X-ray induced foci was characterised by a wide distribution of delays (Supplemental Fig. S6D) thus corroborating our previous results. In conclusion, isolated DSBs show a remarkable variability in their damage response suggesting a delayed conversion of non-DSB lesions into DSBs.

### Delayed occurring DSBs are not due to DNA replication

In order to rule out that the observed delayed NBS1 recruitment is coupled to DSBs generated during replication (e.g. at stalled replication forks), we added a 5-ethynyl-2′-deoxyuridine (EDU)-pulse after irradiation and revisited the nuclei recorded during live cell imaging after fixation and staining of the EDU (S-Phase) and centromere protein-F (CENPF; G2-marker) (Supplemental Fig. S7A). Analysing cell cycle specific EDU and CENPF negative (G1) cells we obtained clear indication of delayed recruiting DSBs. This was evident in both G1-phase as well as S-phase (Supplemental Fig. S7C,D). In addition, the velocity of recruitment starting from the delay time (lag phase subtracted) was found to be nearly identical for G1 and S-phase cells (Supplemental Fig. S7B). In conclusion, as similar recruitment behaviour was observed in G1 phase and S-phase, we demonstrated that the observed delayed DSBs are not replication-dependent.

### Processing of radiation-induced non-DSB lesions contributes to the formation of early responding DSBs

**Figure 6:**
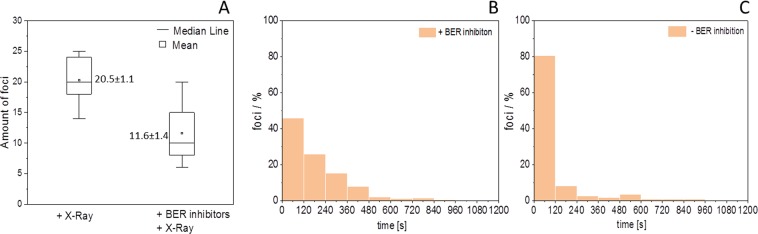
Conversion of clustered non-DSB lesions via base excision-repair (BER) contributes to the DSB load, but is not responsible for the late occurring foci. (**A**) Inhibiting BER by a combination of OGG1 and different APE1 inhibitors prior to irradiation with 0.85 Gy of X-rays substantially decreased the number of formed NBS1-foci in U2OS cells (n = 11 or 21 nuclei per condition, error bars represent 5–95% of data; Value represents mean ± STE). Loss of BER related NBS1 foci formation mainly affects the early recruiting fraction as shown for the distributions of lag-phases in parallel measurements of BER-inhibited (**B**) vs. non- inhibited (**C**) U2OS cells (n = 189 or 108 RIF, respectively). The difference of the distributions is highly significant (Mann-Whitney U-test). Graphs were generated using Origin Pro V.2019 (Originlab Corp., Northampton, MA, USA).

The observation of an individually delayed recruitment of the early DSB repair factors (especially for NBS1) led us to the hypothesis that delayed foci might represent DSBs not generated directly by radiation, but rather through the repair-processing of complex non-DSB lesions. To address the role of damage conversion leading to delayed formation of DSBs, we addressed the influence of BER on the lag phase distribution of late occurring foci by inhibiting core factors. During the course of BER, damaged bases or abasic sites are converted into single-strand breaks (SSBs) by endonuclease activity. In case of clusters of non-DSB lesions or the existence of a SSB in near vicinity on the opposite DNA strand, this conversion may lead to a DSB^25^. The incision step is predominantly done by the apurinic endonuclease APE1, but strand cleavage of the sugar-phosphate backbone can also occur after OGG1 mediated removal of the damaged base via β-elimination at the resulting abasic site^26^. Therefore both reactions were chosen as targets for inhibition. Inhibition of APE1/OGG1 by a combination of methoxyamine hydrochloride and OGG1 inhibitor_O8 reduced the number of observed DSBs after X-rays by around 40%, indicating a major contribution of BER to DSB formation after low-LET radiation exposure. Furthermore, BER inhibition resulted in longer NBS1 foci delays, demonstrating the influence of BER on the average timing of DSB induction and recognition (Fig. 6).

Nevertheless, the observed shift to longer delay times is most probably mainly governed by the loss of early occurring DSBs upon inhibition, indicating that the BER incision is a fast process. In fact, BER is not substantially contributing to the formation of delayed DSBs associated to the prolonged lag-phases of NBS1 recruitment. Rather other mechanisms like conversion of radiation-induced labile sites by post-irradiative chemical modification of sugar lesions^27,28^ may contribute to the conversion of clustered non-DSB lesions. The shift in the lag phase distribution to longer delay times was confirmed by single treatments with the APE1 inhibitor methoxyamine as well as a second APE1 inhibitor (CRT0044876) targeting the nuclease activity of APE1 or by oxoguanine glycosylase inhibition (OGG1 inhibitor_O8). All single treatments led to similar delays in the lag phase distribution of detected DSBs, thus supporting the hypothesis of conversion of clustered base damage into DSBs (Supplemental Fig. S8B-D). However, in contrast to the combination of APE1 and OGG1 inhibitors, single inhibitor treatments did not lead to a significant reduction of radiation-induced number of DSBs. Despite a delayed recognition of DSBs, inhibiting APE1 had no major impact on cellular clonogenic survival, indicating that the delay of DSB detection does not lead to enhanced radiosensitivity and cell death (Supplemental Fig. S8A).

### The BER factor XRCC1 accumulates fast but transiently only on clustered in-track lesions

To further elucidate the contribution of BER in the DDR after high-LET particle irradiation, we followed the real-time recruitment of the repair factor XRCC1 using human fibrosarcoma cells (HT1080) stably expressing EGFP-tagged XRCC1. After irradiation with 1 GeV/u Fe or 750 MeV/u U-ions, a clear, very fast and locally highly confined recruitment to the DNA damage along the trajectories could be observed without significant delay (see Supplemental Movies 5 and 6) similar to the one we described previously in mouse cells^29^. However, besides some weak changes in graininess, no clear off-track foci could be visualised, most probably due to the abundance of base damages and SSBs and the low number of accumulated molecules per lesion (Supplemental Fig. S9,A) complicating the analysis of recruitment to off-track damage. In contrast to the more stable binding of the DSB factors NBS1 and 53BP1, the recruitment of XRCC1 to the massive complex damage inside the track shows a transient accumulation peaking around 10 min after irradiation and subsequently decaying with a half-life of around 10 min (Supplemental Fig. S9B). This clearly demonstrates that not all repair factors become trapped at the massive damage induced by the highly ionising core of the HZE tracks. The transient nature of binding of XRCC1 points either to a displacement of XRCC1 by the DSB repair machinery at damage clusters or to a fast processing of the majority of SSBs and base damage even in case of clustered lesions.

### Inhibition of BER does not affect NBS1 recruitment to clustered in-track DSBs

**Figure 7:**
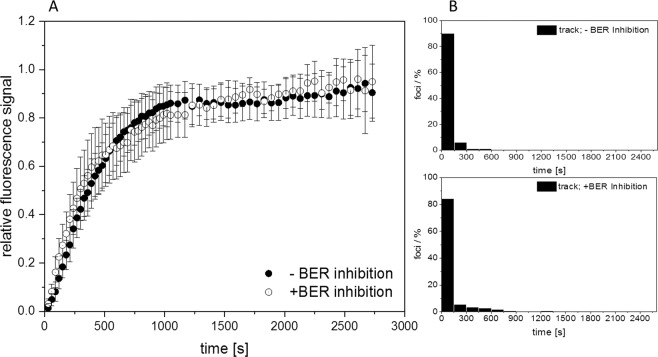
Inhibition of base excision-repair (BER) is not affecting the recruitment kinetics of NBS1-GFP to complex DSBs. (**A**) Inhibiting BER in NBS1-GFP expressing U2OS cells by a combination of OGG1 and APE1 inhibitors prior to irradiation with 350 MeV/u iron ions yielded similar nuclear wide recruitment curves to in track DSBs as in the mock treated control (**A**) (n = 13 or 12 nuclei, respectively). In addition, the lag phase distribution for the start of recruitment of NBS1 to the complex in-track DSBs was similar in both inhibited (B; lower panel) and non-inhibited (B; upper panel) U2OS cells. Both are showing a fast and concerted start of recruitment of NBS1 (n = 236 foci in 10 nuclei or 163 foci in 6 nuclei, respectively). Graphs were generated using Origin Pro V.2019 (Originlab Corp., Northampton, MA, USA).

To address, if BER inhibition generally slows down the recruitment of NBS1 to DSBs, we analysed the recruitment kinetics at the clustered in-track DSBs. Whereas inhibition of the incision step of BER clearly affected the recruitment to the isolated DSBs induced after X-rays, no change in the slope or distribution of delay-times was obtained for in-track RIF after irradiation with 350 MeV/u Fe-ions (Fig. 7). This indicates that the recruitment to the complex damage in the track is mainly governed by the high number of promptly induced DSBs and that the application of the BER inhibitors is not changing the general kinetics of NBS1.

## Discussion

In this study we measured simultaneously real time recruitment kinetics of early DDR factors to clustered and simple DSBs in the same cell upon traversal by high-LET charged particles. Simple, off-track DSBs produced by δ-electrons of these particles have been predicted using biophysical modelling^30^, but hitherto have been studied only in fixed samples^31–33^, unavoidably missing the early activation of repair factors and the dynamics of individual foci. The complex, clustered DSBs formed along the core of the traversing HZE ion showed a prompt accumulation of the early DSB binding factor NBS1, in line with our previous measurements with very densely ionising low energy particles characterised by fast kinetics of NBS1 at the tracks due to fast ATM activation^19^. For both types of high–LET particles local doses in the range of kGy are deposited along the core leading to the generation of complex clustered DSBs with multiple DSBs in each focus^20,31^. The analysis of NBS1 accumulation at individual simple, off-track DSBs revealed a significant fraction of lesions showing a delayed onset of recruitment as well as a more transient binding to the damage. This observation was rather independent from the applied off-track dose as revealed by the usage of different HZE particles. Of note, real time measurements of repair factors, even those acting early in the DSB response, might not reflect the exact timing of DSB induction and repair in case some pre-processing at the damage site was needed. However, even larger scale chromatin remodelling after particle irradiation in heterochromatin has been shown to occur in the order of tens of seconds and will thus not delay the recruitment of early repair factors to a larger degree^29,34^. These general observations were confirmed using charged particles of varying atomic number and energy as well as 53BP1-RIF formation, a surrogate marker of DSBs^18^ like γH2AX. Compared to γH2AX or 53BP1, NBS1 requires less pre-processing steps before the onset of recruitment. Thus the observation of partially delayed recruitment of both NBS1 and 53BP1 to individual DSBs corroborates our interpretation of an individually delayed DSB formation rather than local differences in protein activation. The shorter persistence of the protein accumulation at the simple, δ-ray-induced off-track DSBs reflects a faster processing and repair compared to the clustered DSB induced along the track core. This interpretation is consistent with experiments showing impaired or delayed repair at complex lesions^14,21,29,32,33,35,36^ or increased accretion of 53BP1 to DSB-clusters versus single-DSBs^37^. Stalled repair clusters may persist for a substantial time in cells and lead to chromosomal breakage^11^ and enhanced mutagenesis^38^.

The individually delayed recruitment of the off-track RIF produced by electrons emitted by fast heavy ions was similarly observed in RIFs produced by low-LET X-rays (Fig. 5 und Supplemental Fig. S6), indicating that both radiation types induce similar damage spectra including non-DSB clustered lesions. This is expected from the physical process of energy deposition by electrons produced by either a photon (X-rays) or an HZE ion (δ-rays) atomic ionisation^30^. In line with that notation, we have found no correlation between the lag times and the radial distance from the core for the off-track DSBs, suggesting that most δ-ray and X-ray induced DSBs are generated by the high energy deposition of the electrons toward the end of their range. Protein accumulation and release from RIF are similar for X- and δ-rays (Fig. 5), corroborating the evidence that the complexity of DSBs determines their repair kinetics. Compared to the off-track areas or after application of sparsely ionising radiation, where DSBs are produced mainly by indirect, radical-mediated action, a higher fraction of direct damage is induced inside the densely ionising ion tracks^39,40^. Nevertheless, these differences will not impact on the observed delay times as the timescale of the physico-chemical propagation of the highly reactive radicals are much shorter (µs) than the recruitment kinetics addressed in this study.

The contribution of BER-processing to the formation of DSBs after irradiation was demonstrated here by preventing the incision step of the DNA backbone via inhibition of the endonuclease activity, thus limiting the generation of SSBs. It has been shown that two opposite base lesions >3 bp apart or combinations of SSBs and base damage/loss results in DSB formation during the BER excision/ incision step^27,41,42^. The reduction in the number of early recruiting RIFs observed in our study is in agreement with a fast operation of BER incision on lesions containing 2 AP sites as bi-stranded cluster^38^ or other combinations of clustered non-DSB lesions which can be efficiently converted into DSBs^41^. Our results suggest that besides participation of BER in DSB production, this pathway is not responsible for the observed delayed recruiting fraction of DSBs. The inhibition of the early steps of BER had no significant impact on the recruitment of NBS1 to in-track RIF, indicating that the severe clustered damage in the high-LET core is dominated by prompt DSBs, which already activate the local chromatin repair response independently of BER.

Delayed NBS1 recruitment was observed throughout the cell cycle in interphase cells (Supplemental Fig. S6), thus ruling out replication-dependent conversion of non-prompt DSBs^43^ as a major source for late responding RIFs. We therefore propose that other clusters of radiation-induced labile non-DSB lesions are responsible for our observation. Heat-labile sites have been described as source of DSBs analysed using pulse-field gel electrophoresis^27^, but it has been shown that a subset of these can be converted into DSB also under physiological conditions in cells, thus leading to the generation of delayed occurring DSBs^27^.

In conclusion, our study shows that DDR kinetics is strongly influenced by damage complexity. While additional factors like the local chromatin environment (e.g. heterochromatin^44^) can contribute to differences as well, we propose that enzymatic processing and conversion of non-DSB lesions play major roles in the repair kinetics of simple DSBs. Damage induced by isolated electrons is processed with similar mechanisms, independently of their source (photons or charged particles). Recruitment of DNA repair proteins is faster to clustered, complex DNA lesions and dominated by prompt DSB formation which can be repaired without involvement of BER. The current mechanistic models of radiation action, aiming to link DNA damage and repair to late cellular effects^45–50^, currently use mostly protein recruitment kinetics from laser-induced DNA lesion experiments^51,52^ or the in-track RIF produced by heavy ions^19,51,53^, thus neglecting the damage induced by the δ-rays, which transport roughly 50% of the energy deposited by the primary ion^54^. Our results provide the necessary data to treat separately simple and clustered DNA in biophysical modelling of radiation action.

## Materials and Methods

### Cell culture

Human osteosarcoma cells U2OS stably expressing NBS1-GFP^55^ or 53BP1-GFP^56^ (kindly provided by Claudia Lukas Danish Cancer Society, Copenhagen, Denmark) and human fibrosarcoma cells HT1080 stably expressing EGFP-XRCC1^11^ (kindly provided by David Chen, UT Southwestern, Dallas, USA) were cultured in Dulbecco’s Modified Eagle Medium (DMEM) with 4,5 g/L D-glucose supplemented with 10% (v/v) fetal bovine serum (FCS) (both from Biochrom AG, Berlin, Germany), 1 µg/ml Puromycin (Sigma-Aldrich) and for U2OS-NBS1-GFP cells culture medium additionally contained 400 µg/ml G418 (AdipoGen Life Sciences). For microscopic experiments cells were cultured similarly but using cell culture medium deprived of phenol red. The cells were kept at 37 °C under a humidified atmosphere with 5% CO_2_.

### Cell irradiation

For ion irradiation 70 000 cells/mL were seeded 24 h prior to experiments on 4-well chamber slides (Thermo Scientific), for X-Ray irradiation 75 000/mL cells on petri dishes with glass bottom (35 mm Ø; 10 mm thickness; Greiner Bio-One GmbH). In the experiment performing retrospective cell cycle analysis after irradiation, cells were seeded on petri dishes with a grid-500 etched glass bottom (35 mm Ø; 0,17 mm thickness; Ibidi, Germany) to be able to revisit cells after fixation. Charged particle irradiation was performed using a horizontal beam (fluence 5·10^6^ cm^−2^) at the heavy-ion synchrotron (SIS) of the GSI Helmholtz Centre for Heavy Ion Research using different HZE species ^56^Fe (1 GeV/u; LET 150 keV/µm, 1.2 Gy or 350 MeV/u; LET 220 keV/µm, 1.8 Gy), ^238^U (750 Me V/u; LET 2020 keV/µm, 16 Gy), ^59^Ni (350 MeV/u; LET 255 keV/u, 2.0 Gy) as indicated. Roughly a fraction of half of the dose can be considered to be delivered to the track centre and penumbra, respectively. Cell irradiation with X-Ray was performed as described before^57,58^. Briefly, a 35 kV X-ray tube (GE Inspection Technology, Ahrensburg, Germany) which was operated at 80 mA delivering a dose rate of 0.85 Gy/s at the cell layer was used. Dosimetry was done using a Unidos dosemeter equipped with a soft X-ray ionisation chamber TM23342A (PTW, Freiburg, Germany) applying a correction factor (1.2) for the dose increase at the glass surface. For inhibition of BER pathway methoxyamine hydrochloride (20–35 mM)^59^, OGG1 inhibitor_O8 (50–75 µM)^60^ and CRT0044876 (150–240 µM)^61^ (all purchased from Sigma-Aldrich) were incubated with cells 1–2 h prior to irradiation as indicated.

### Live cell imaging and data analysis

Live cell observations were performed using a modified remote controlled OLIMPUS IX71 or IX73 microscope for both ion and X-ray irradiation using an UPlanFL60x/1.2 water or 100 × /1.4 oil immersion lens and a 1.6 x optovar as described in^19^. Except for the experiment using the 350 MeV/u Fe beam, where an Andor Zyla 4.2 sCMOS camera was used, image acquisition was done with an EM-CCD camera type DU-888 (Andor Technology, Belfast, Ireland). For time lapse imaging of z-stacks, AndorIQ software was used. Fluorescence was excited with the CoolLED system or monochromator Polychrome V (TILL Photonics GmbH, Gräfelfing, Germany), respectively. Acquired z-stacks (0.5 μm steps) were deconvoluted using the Huygens essential software (SVI, Netherlands). Image analysis of nuclear wide recruitment kinetics was performed with the software package ImageJ (https://imagej.nih.gov/ij/; NIH, USA) as described in^19^. To facilitate single focus analysis, a software package (ImageD - Version 1_6–8 with plugin *Foci Tracking*) was developed in house. Here, foci were automatically detected in maximum projections of the z-stacks using locally adaptive thresholds with the option of manual corrections. The automatic evaluation of lag phases is based on the variation in the standard deviation between the selected time windows with relative fluorescence intensity of the lag time ≥10% of the maximal intensity value of the concerned focus as an additional parameter. The measurements were double normalised to the signal loss during the image acquisition and to the plateau, according to^19^ yielding the relative fluorescence signal. Cell motion during acquisition was compensated with the ImageJ StackReg plugin (Philippe Thevenaz, Lausanne, Switzerland). Data analysis was done using Excel 2010 (Microsoft Corporation, USA) or Origin Pro V.2019 (Originlab Corp., Northampton, MA, USA). For figure grouping, Powerpoint 2010 (Microsoft Corporation, USA) was used.

### Immunocytochemistry

To determine the cell cycle phase U2OS-NBS1-GFP cells were treated 15 min with 10 µM EdU (5-ethynyl-2′-deoxyuridine; EdU Click-it kit 647 from Carl Roth) directly after live cell microscopy was finished. Cells were fixed 15 min in 2% formaldehyde in PBS (prepared from PFA, Carl Roth, Karlsruhe) and permeabilised with 0.5% Triton-X (Applichem, Darmstadt) for 10 min at room temperature. To block unspecific binding sites 0.4% BSA (Carl Roth, Karlsruhe) was added for 20 min. As primary antibody polyclonal rabbit CENP-F (1:750; Novus Biologicals) and as secondary antibody Alexa 647 donkey anti-rabbit (1:400; Life Technologies) were used. DNA was counterstained with a 4′,6-diamidino-2-phenylindole (DAPI; Labor-Service, Griesheim) solution of 1 µg/ml in PBS for 15 min. Samples were mounted in Vectrashield Mounting Medium (Vector Laboratories, Burlingame, U.S.A.). The samples were analysed using a Leica TCS SPE microscope and LAS software (both Leica Mikrosysteme, Wetzlar). The respective excitation and emission wavelengths were 350/470 nm for DAPI, 650/665 nm for EDU and 578/603 nm for CENP-F.

### Statistical analysis

Statistical significance was determined by two-tailed Student’s t-test or Mann–Whitney rank-test. Values of P < 0.05 were considered statistically significant. Data are represented as means ± 95% CI, as box plots showing the 25/75 quartiles and 5–95% whiskers or as frequency distributions (histograms) as indicated.

## Supplementary information

Supplementary Information.

Supplementary Information2.

Supplementary Information3.

Supplementary Information4.

Supplementary Information5.

Supplementary Information6.

Supplementary Information7.

## Data Availability

The data that support the findings of this study are available from the corresponding author on reasonable request.
