# Supplementary material for: Differential Repair Protein Recruitment at Sites of Clustered and Isolated DNA Double-Strand Breaks Produced by High-Energy Heavy Ions
Source: Sci Rep. 2020 Jan 29;10:1443. doi: 10.1038/s41598-020-58084-6 (PMC6989695; doi:10.1038/s41598-020-58084-6)
Supplement: Supplementary file 1 — Supplementary Information. [file 41598_2020_58084_MOESM1_ESM.pptx]

## Slide 1
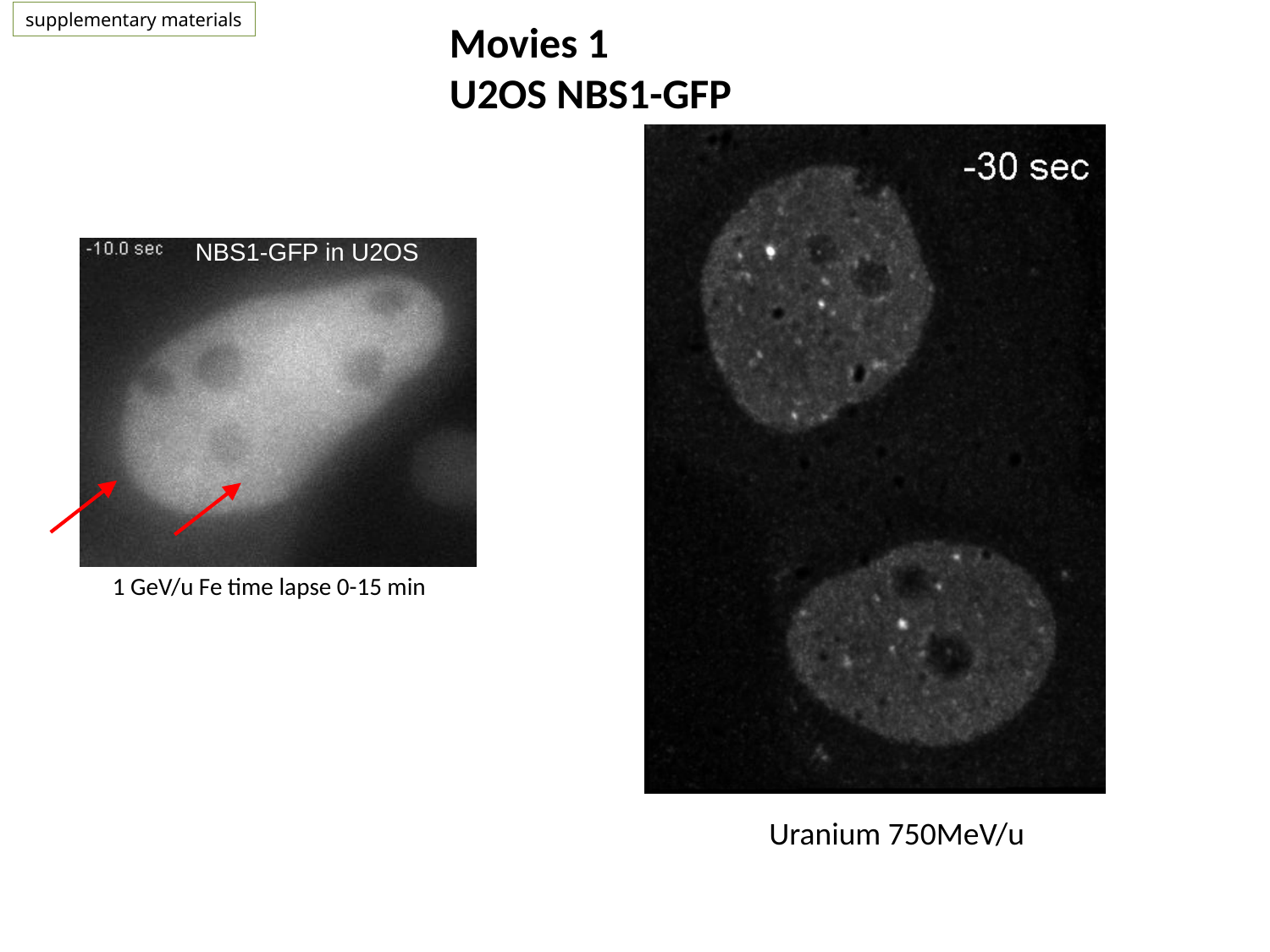

supplementary materials
Movies 1
U2OS NBS1-GFP
NBS1-GFP in U2OS
1 GeV/u Fe time lapse 0-15 min
Uranium 750MeV/u
