# Supplementary figures and images for: Differential Repair Protein Recruitment at Sites of Clustered and Isolated DNA Double-Strand Breaks Produced by High-Energy Heavy Ions
Source: Sci Rep. 2020 Jan 29;10:1443. doi: 10.1038/s41598-020-58084-6 (PMC6989695; doi:10.1038/s41598-020-58084-6)

## Slide 1
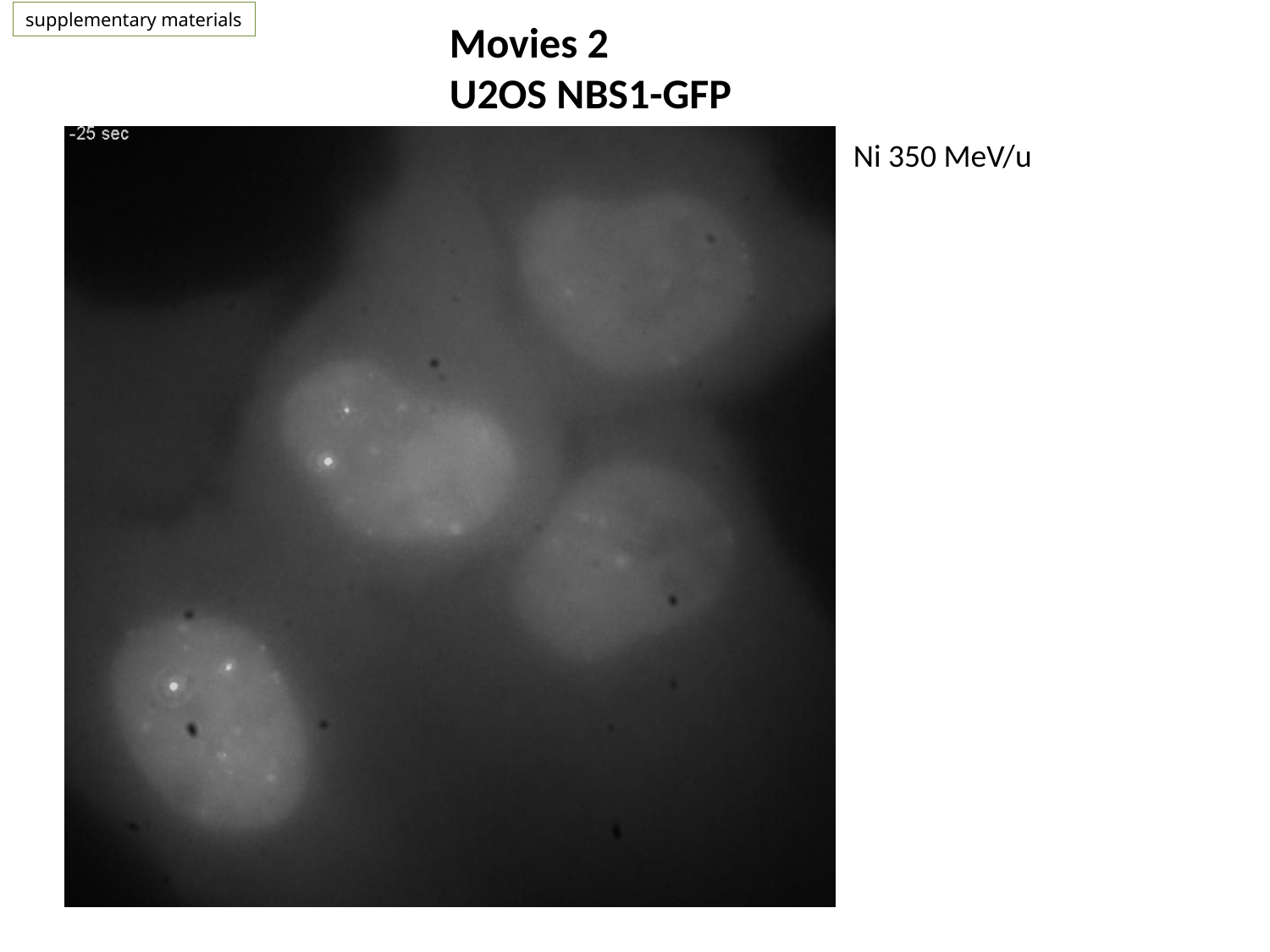

supplementary materials
Movies 2
U2OS NBS1-GFP
Ni 350 MeV/u

Supplement: Supplementary file 2 — Supplementary Information2. [file 41598_2020_58084_MOESM2_ESM.pptx]

## Slide 1
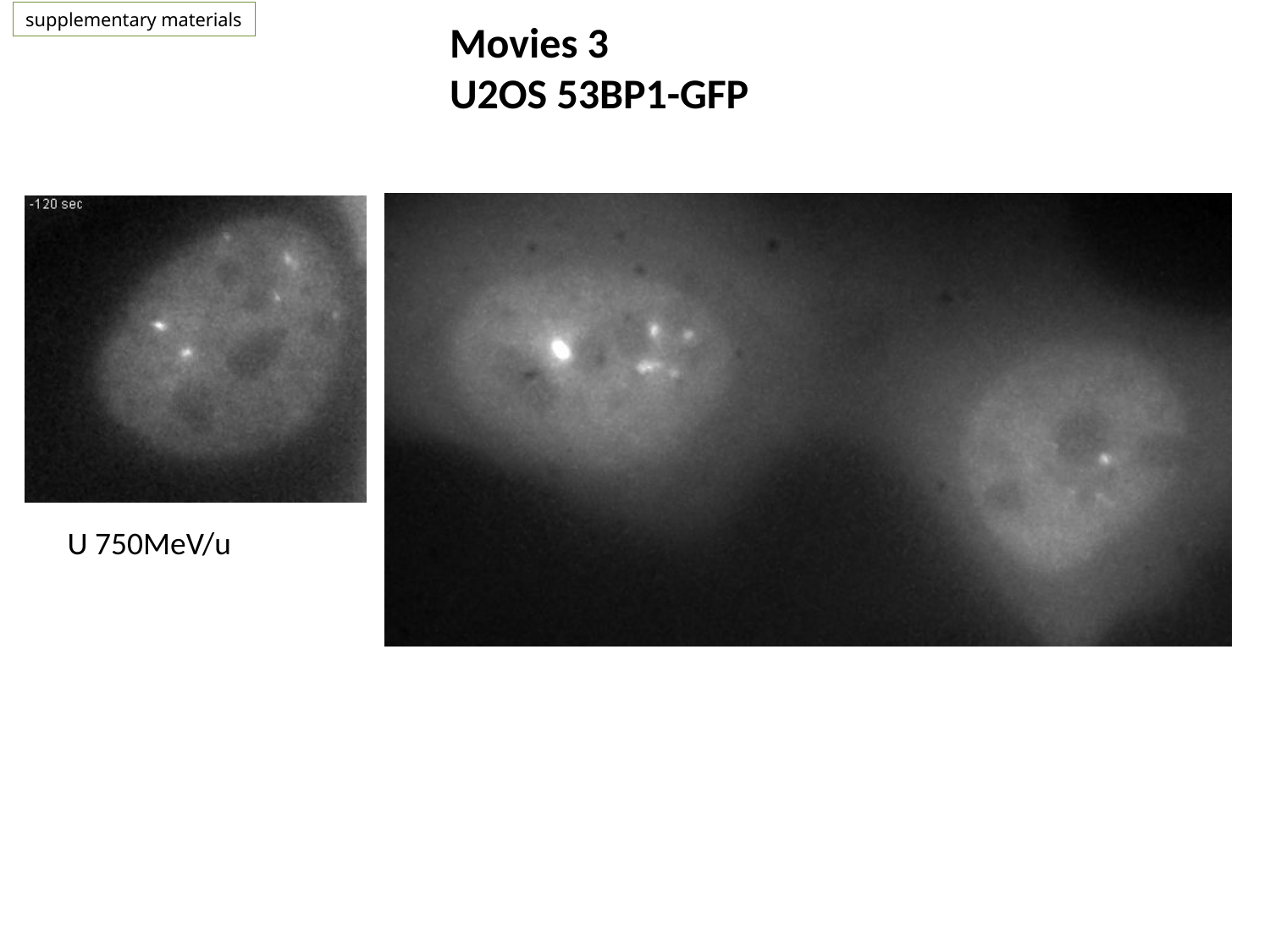

supplementary materials
Movies 3
U2OS 53BP1-GFP
U2OS-53BP1_GFP + 0.7 Gy
U 750MeV/u

Supplement: Supplementary file 3 — Supplementary Information3. [file 41598_2020_58084_MOESM3_ESM.pptx]

## Slide 1
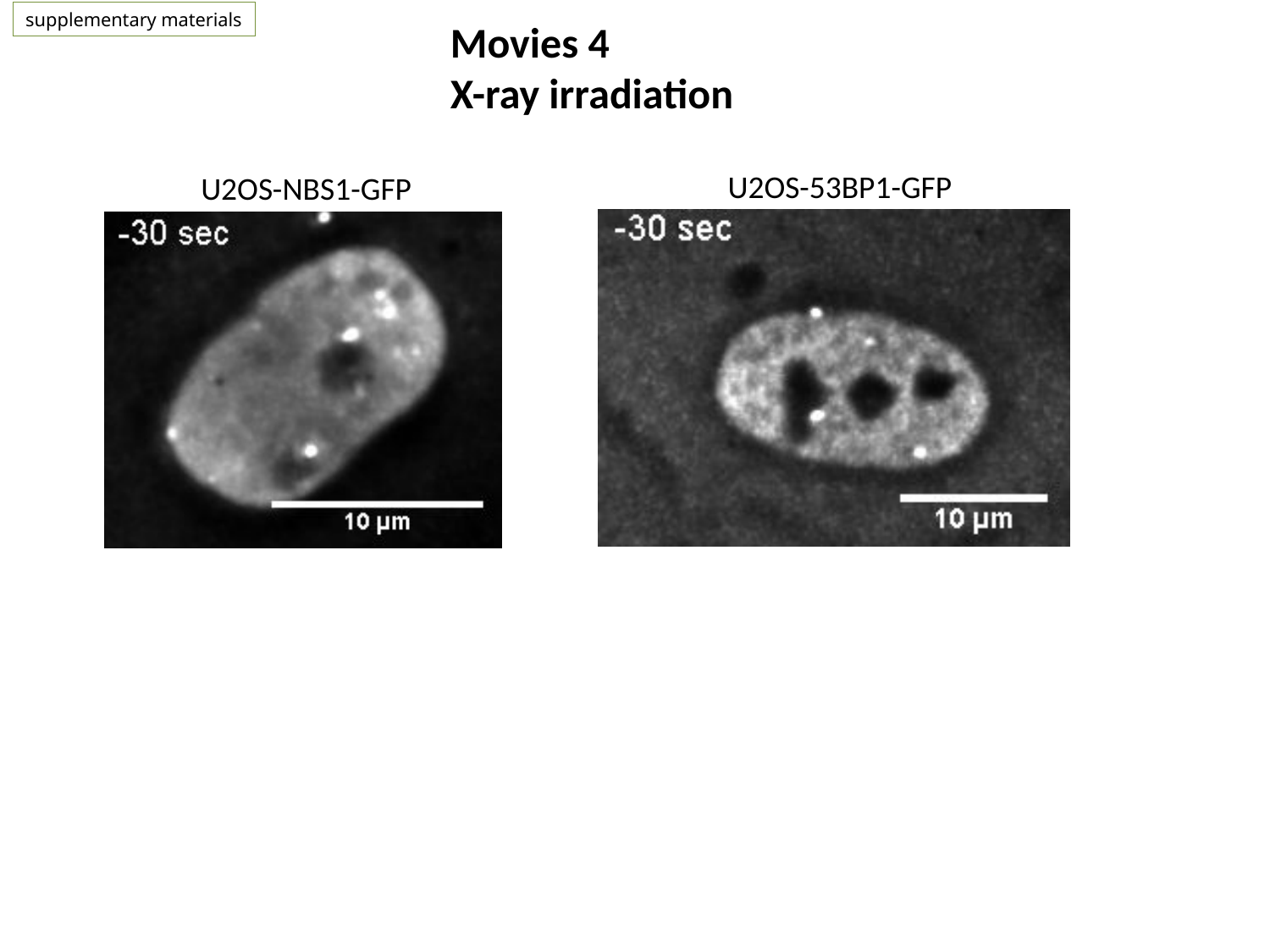

supplementary materials
Movies 4
X-ray irradiation
U2OS-53BP1_GFP + 0.7 Gy
U2OS-53BP1-GFP
U2OS-NBS1-GFP

Supplement: Supplementary file 4 — Supplementary Information4. [file 41598_2020_58084_MOESM4_ESM.pptx]

## Slide 1
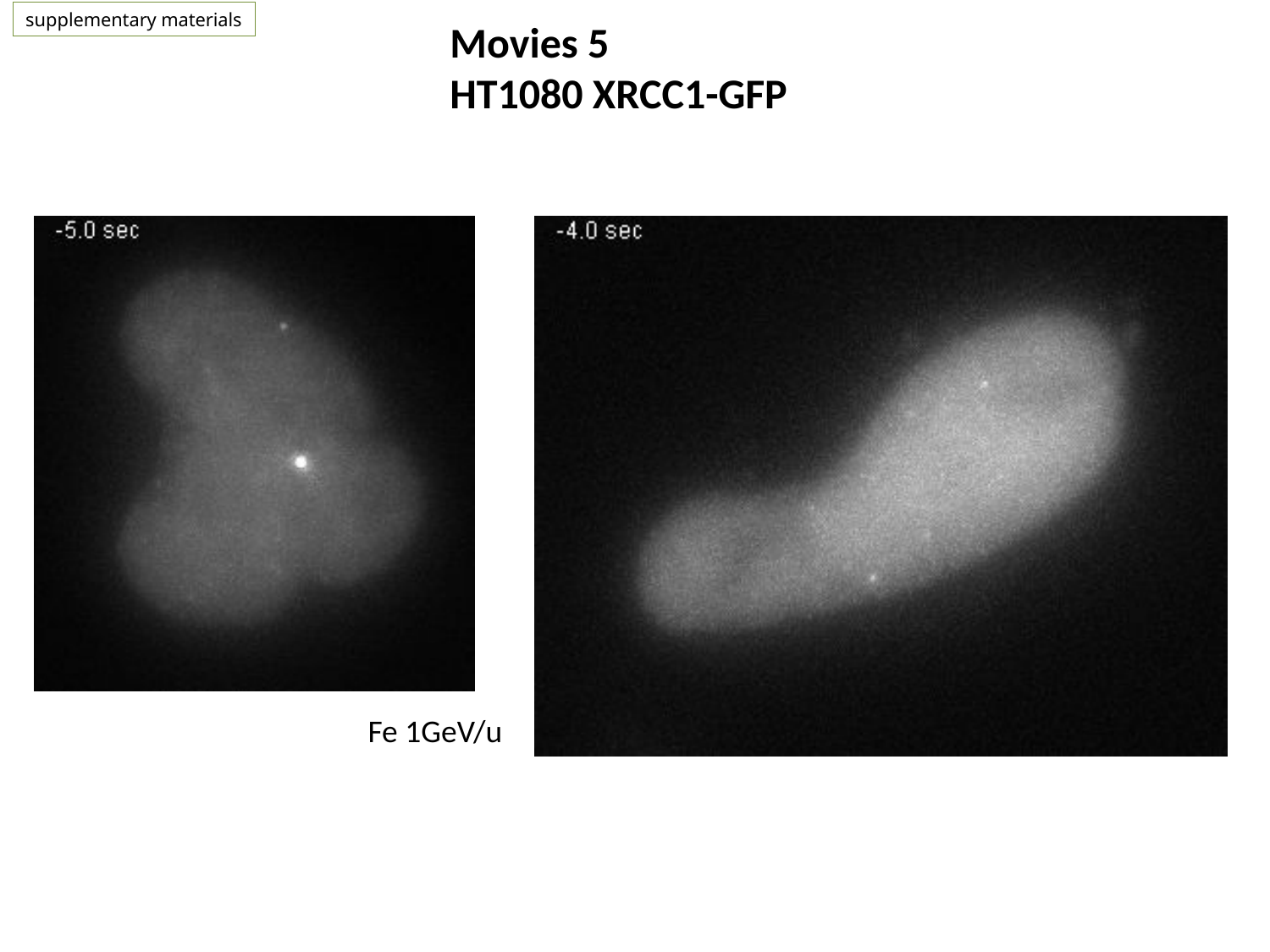

supplementary materials
Movies 5
HT1080 XRCC1-GFP
U2OS-53BP1_GFP + 0.7 Gy
Fe 1GeV/u

Supplement: Supplementary file 5 — Supplementary Information5. [file 41598_2020_58084_MOESM5_ESM.pptx]

## Slide 1
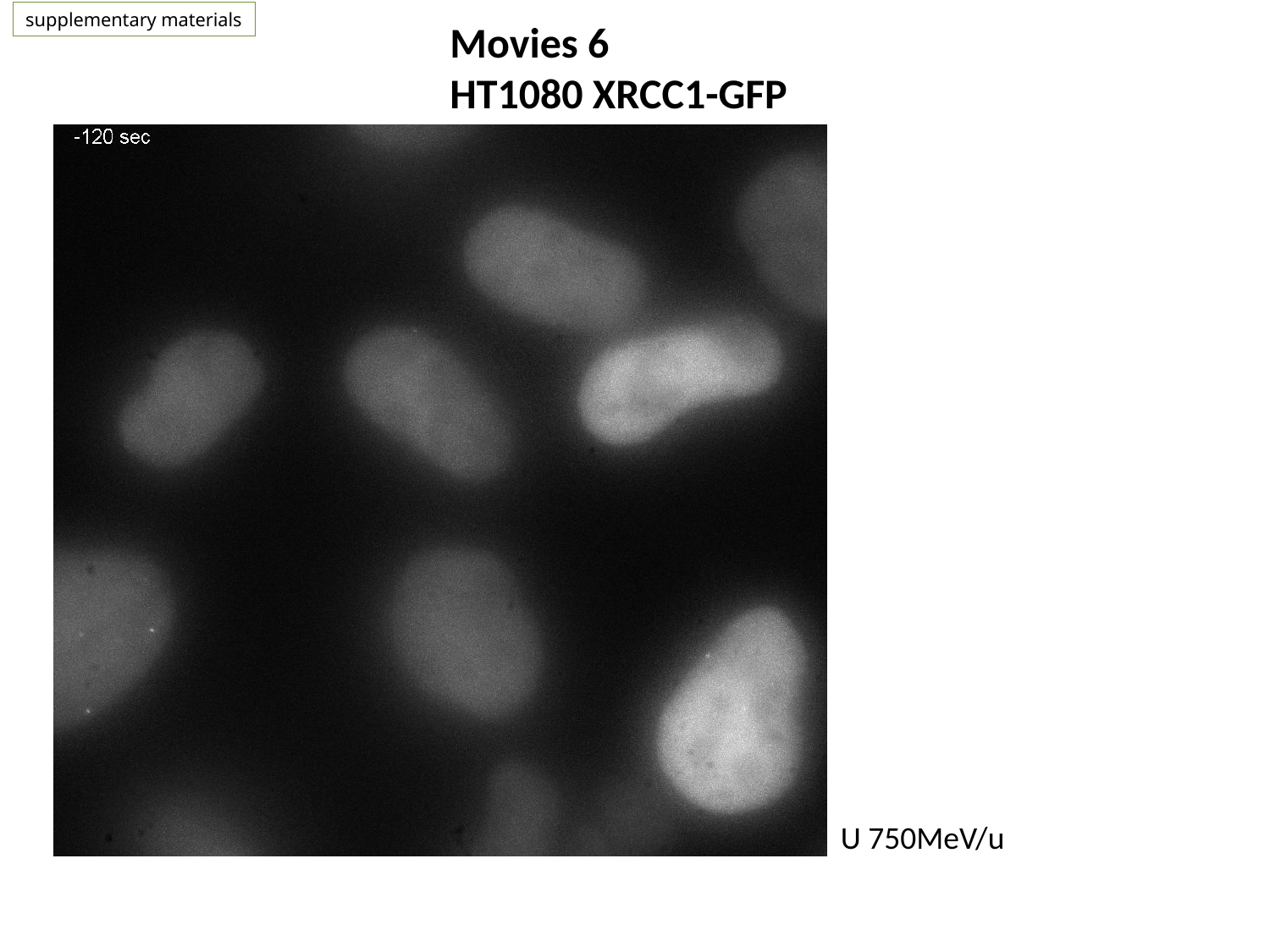

supplementary materials
Movies 6
HT1080 XRCC1-GFP
U2OS-53BP1_GFP + 0.7 Gy
U 750MeV/u

Supplement: Supplementary file 6 — Supplementary Information6. [file 41598_2020_58084_MOESM6_ESM.pptx]
