# Supplementary material for: Differential Repair Protein Recruitment at Sites of Clustered and Isolated DNA Double-Strand Breaks Produced by High-Energy Heavy Ions
Source: Sci Rep. 2020 Jan 29;10:1443. doi: 10.1038/s41598-020-58084-6 (PMC6989695; doi:10.1038/s41598-020-58084-6)
Supplement: Supplementary file 7 — Supplementary Information7. [file 41598_2020_58084_MOESM7_ESM.pdf]

Supplemental Figures

Differential repair protein recruitment at sites of clustered and isolated DNA double-strand breaks produced by high-energy heavy ions

Burkhard Jakob[1,] Monika Dubiak-Szepietowska[1], Ellen Janiel[1,2], Alina Schmidt[1,2], Marco Durante[1,3]& Gisela Taucher-Scholz[1,2]
[1]Department of Biophysics, GSI Helmholtzzentrum für Schwerionenforschung, 64291 Darmstadt, Germany. [2]Department of Biology , Technische Universität Darmstadt, 64287 Darmstadt, Germany. [3]Department of Physics, Technische Universität Darmstadt, 64287 Darmstadt, Germany. Correspondence and requests for materials should be addressed to B.J. (email: b.jakob@gsi.de) or to M.D. (email: m.durante@gsi.de).

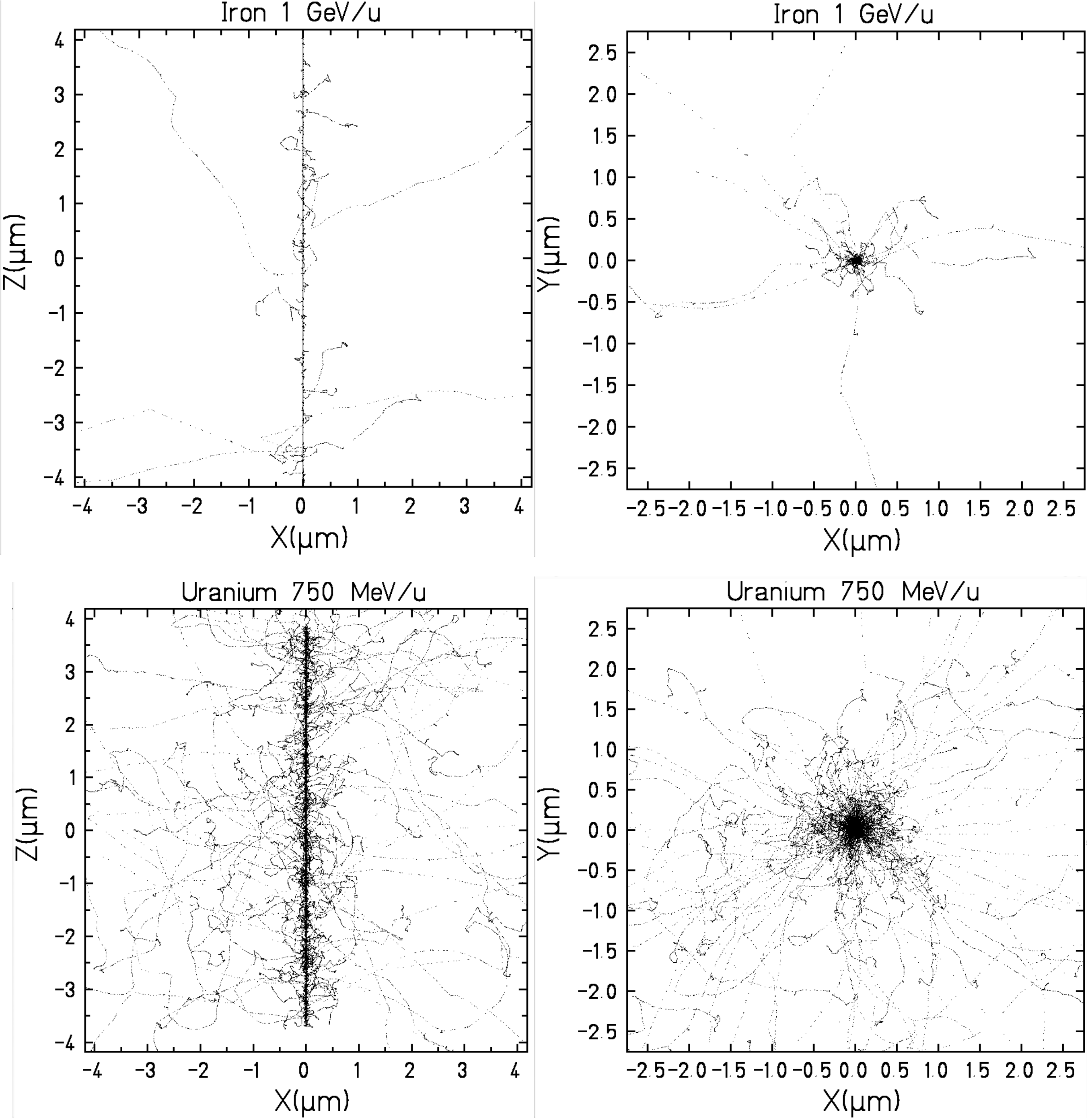

Figure S1. Monte Carlo simulation of the HZE particles used in this experiments. The simulation shows a single ^{56}Fe and ^{238}U moving along the Z-axis and hitting orthogonally the XY-plane. Each dot represent a ionization in the water medium. The dense region along the particle traversal (core) indicates ionization clusters due to the high linear energy transfer (LET) of the ion and is responsible for clustered DSBs. High-energy electrons (δ-rays) are sparsely ionizing (similar to electrons produced by X-rays) and induce mostly simple DSB in a DNA target. U-ions have higher LET than Fe and they produce more δ-electrons per unit track length and a wider core around the line of traversal. The maximum range in water of the δ-rays depends only on the HZE velocity and is ~ 7.8 mm for Fe-ions and ~ 4.8 mm for U-ions at these velocities. However, the density of the electrons is very low at those large distances, an the dose decrease as r^{-2} with the distance r from the track core. Simulation by the Monte Carlo code TRAX developed at GSI.

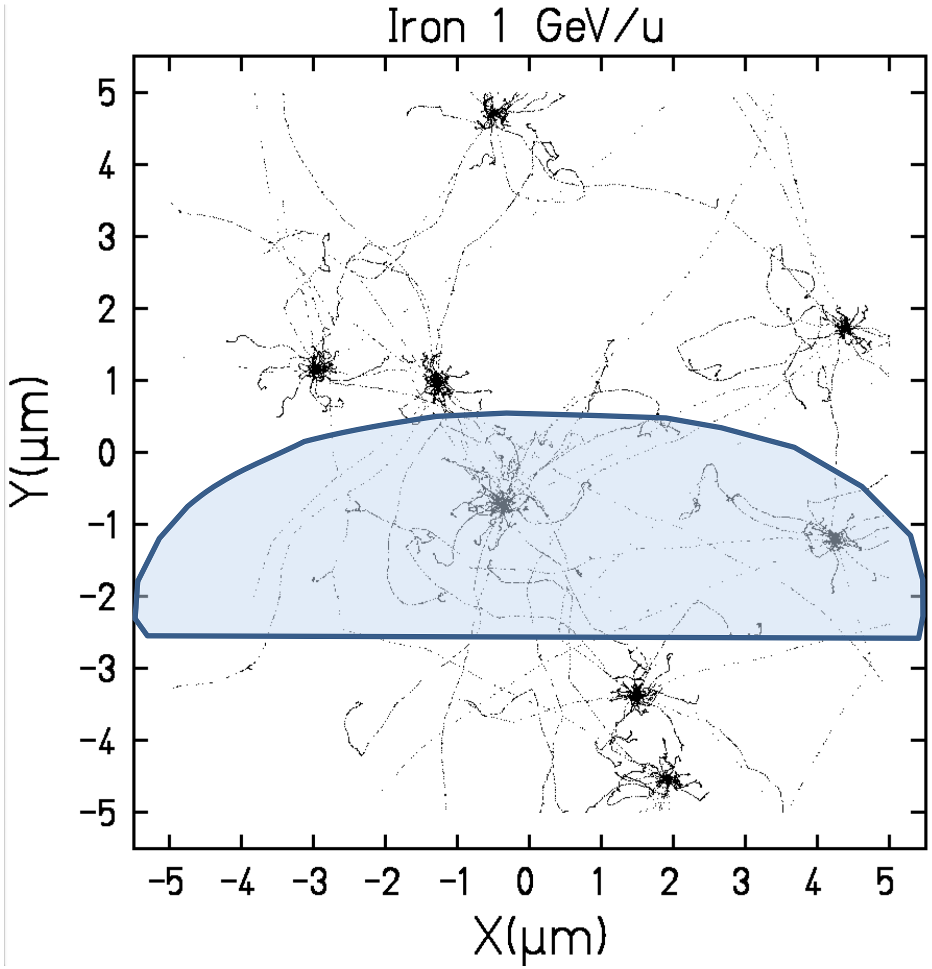

Figure S2. Monte Carlo simulation of the HZE ion tracks in our experiments.
At a fluence of $5 \cdot 10^6$ particles/cm^{-2} used in our experiments, δ-rays from different tracks can overlap thus increasing the probability of scoring off-track RIF due to simple DSBs induced by δ-electrons. In this Monte Carlo (TRAX-code) simulation the δ-ray overlap is shown for Fe 1 GeV/u ions on a 10x10 μm^2 target on the XY-plane at the fluence used in our experiments. The blue overlay represents a schematic of a horizontal cross section of typical flattened cell nucleus in cell monolayer experiments. Simulation by the Monte Carlo code TRAX developed at GSI.

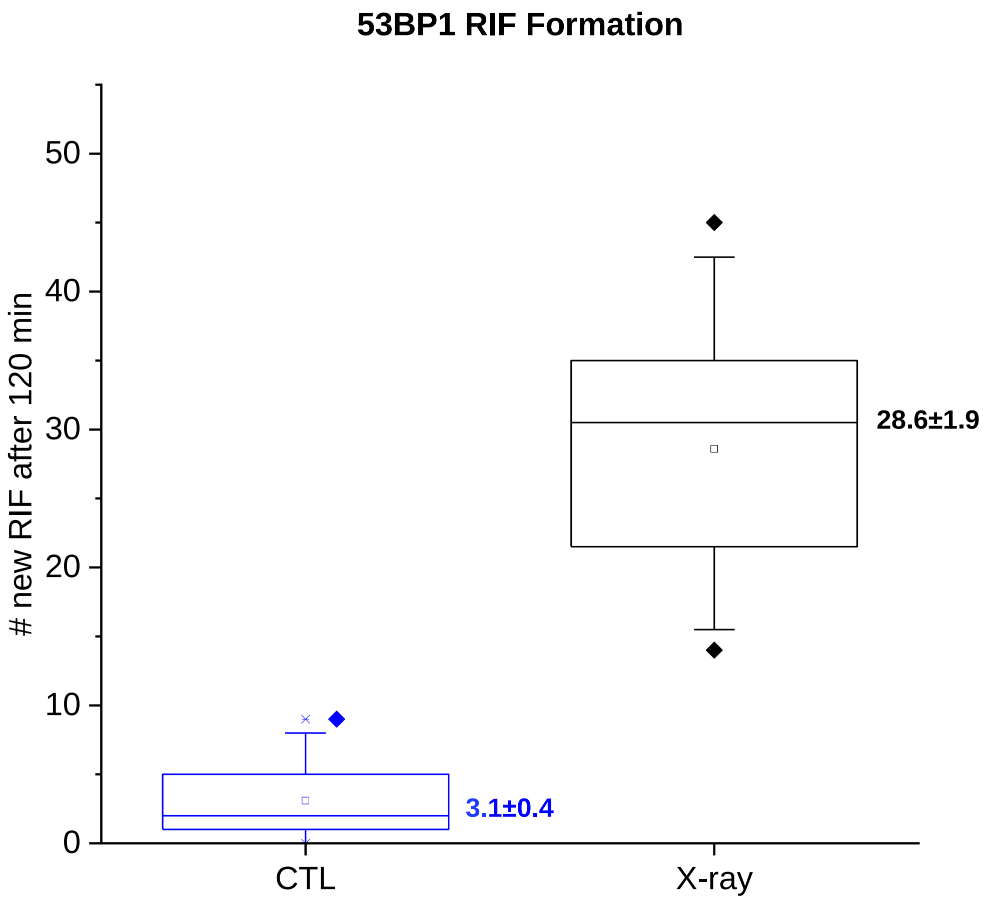

Figure S3. Majority of DSBs is induced by irradiation and not by microscopy procedure. Mock irradiated cells only showed relatively few 53BP1-GFP RIF during 2h imaging under identical conditions in comparison to irradiation with 0.85 Gy of x-rays leading to a rate of 1-2 RIF/h. Error bars represent 5-95% of data. Value represents mean±STE after 2h; n=31 nuclei for control and n=20 for x-rays). For NBS1-GFP no foci equally to irradiation induced IRIFs could be detected in same irradiated samples (data not shown). These measurements are indicating that delayed occurring foci are not induced by the illumination during the course of image acquisition. Graph was generated using Origin Pro V.2019 (Originlab Corp., Northampton, MA, USA).

A

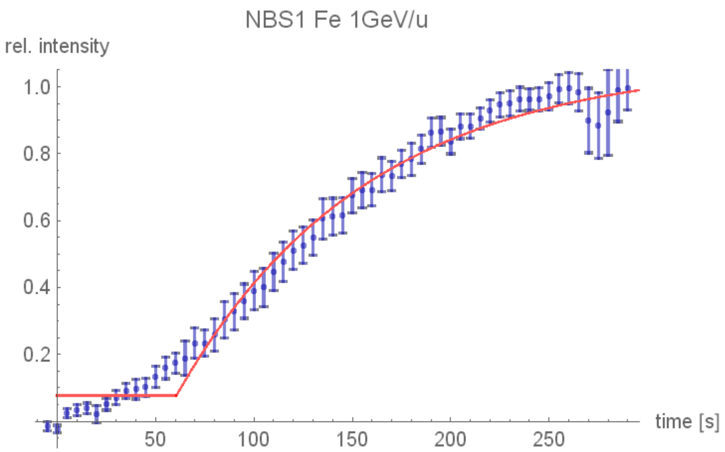

	"Estimate"	"Asymptotic SE"	"CI"
tlag	60.6	2.2	{56.14,64.98}
t1	96.1	4.2	{87.68,104.62}
C	0.076	0.011	{0.054,0.099}

B

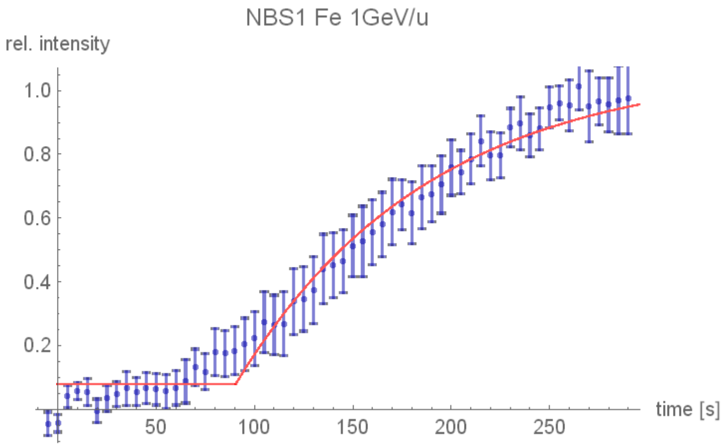

	"Estimate"	"Asymptotic SE"	"CI"
tlag	90.4	2.6	{85.15,95.64}
t1	97.5	4.7	{88.12,106.87}
C	0.078	0.011	{0.056,0.101}

Figure S4. Mathematical description of delayed recruiting NBS1 after irradiation with 1GeV/u Fe-ions. Fluorescence intensity date from Fig. 2A were fitted by the following piecewise function using Mathematica (V10.4 Wolfram Research, Hanborough Oxfordshire, UK): Piecewise[{{C, t <= tlag}, {1*((1 - Exp [(-t + tlag)/t1])) + C, t > tlag}}]. Error bars represent 95% CI. (A) In-track RIF, (B) Off-track RIF. Table shows fitting results with standard error "SE" and confidence intervall "CI".

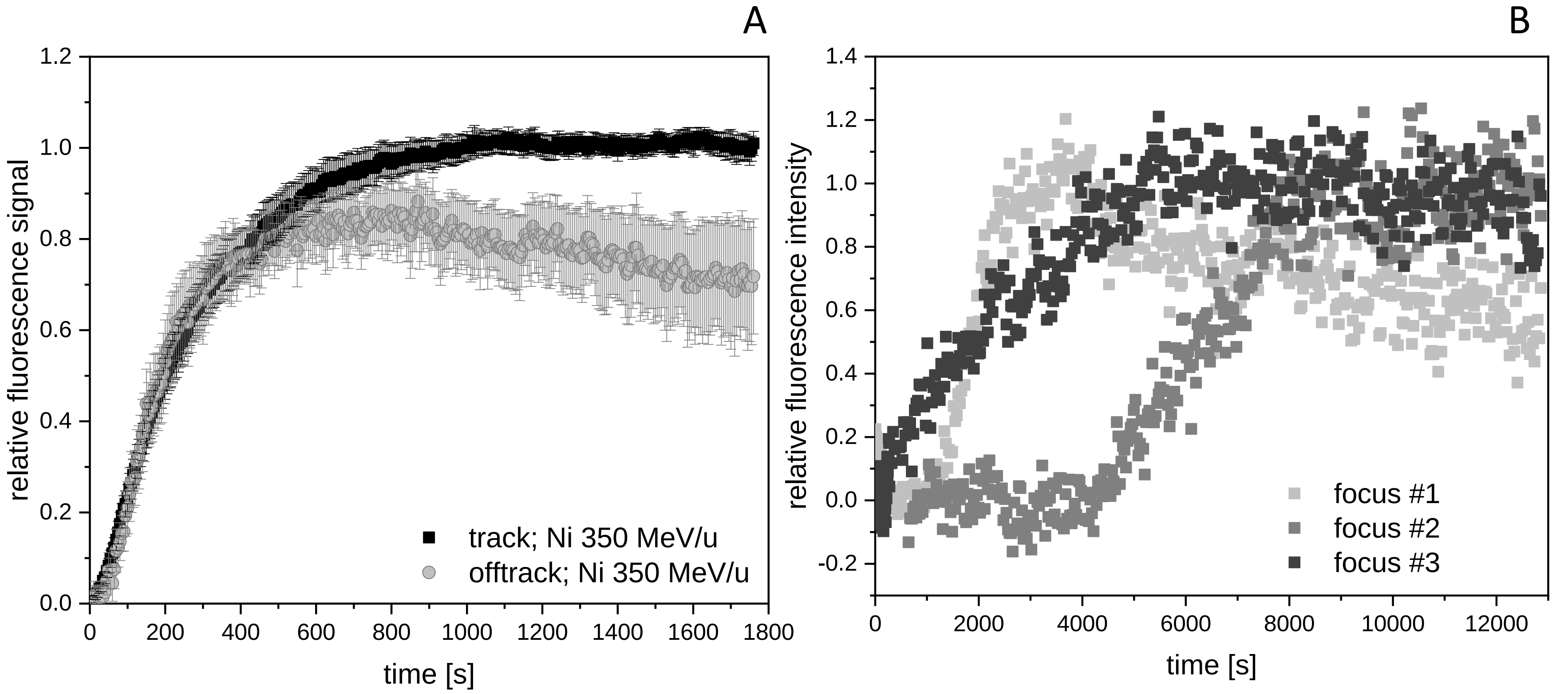

Figure S5. (A) Off track DSB showing early recruitment of NBS1 display transient binding. Nuclear wide NBS1-GFP recruitment kinetics of early recruiting RIF after irradiation of U2OS-cells with 350MeV/u Ni-ions (n=35 nuclei for both, Error bars 95% CI). Similar to the irradiation with Uranium-ions (Fig. 2 and 3) recruitment to these off-track DSB was transient with a maximum at around 10-15 min. **(B) Similar to Uranium, individually delayed NBS1 foci formation can also be observed for other HZE particles.** Analysis of NBS1 recruitment to individual off-track DSBs during the whole course of the time-lapse measurement revealed the occurrence of delayed detected DSBs for 350 MeV/u Ni-ions. Graphs were generated using Origin Pro V.2019 (Originlab Corp., Northampton, MA, USA).

supplementary materials

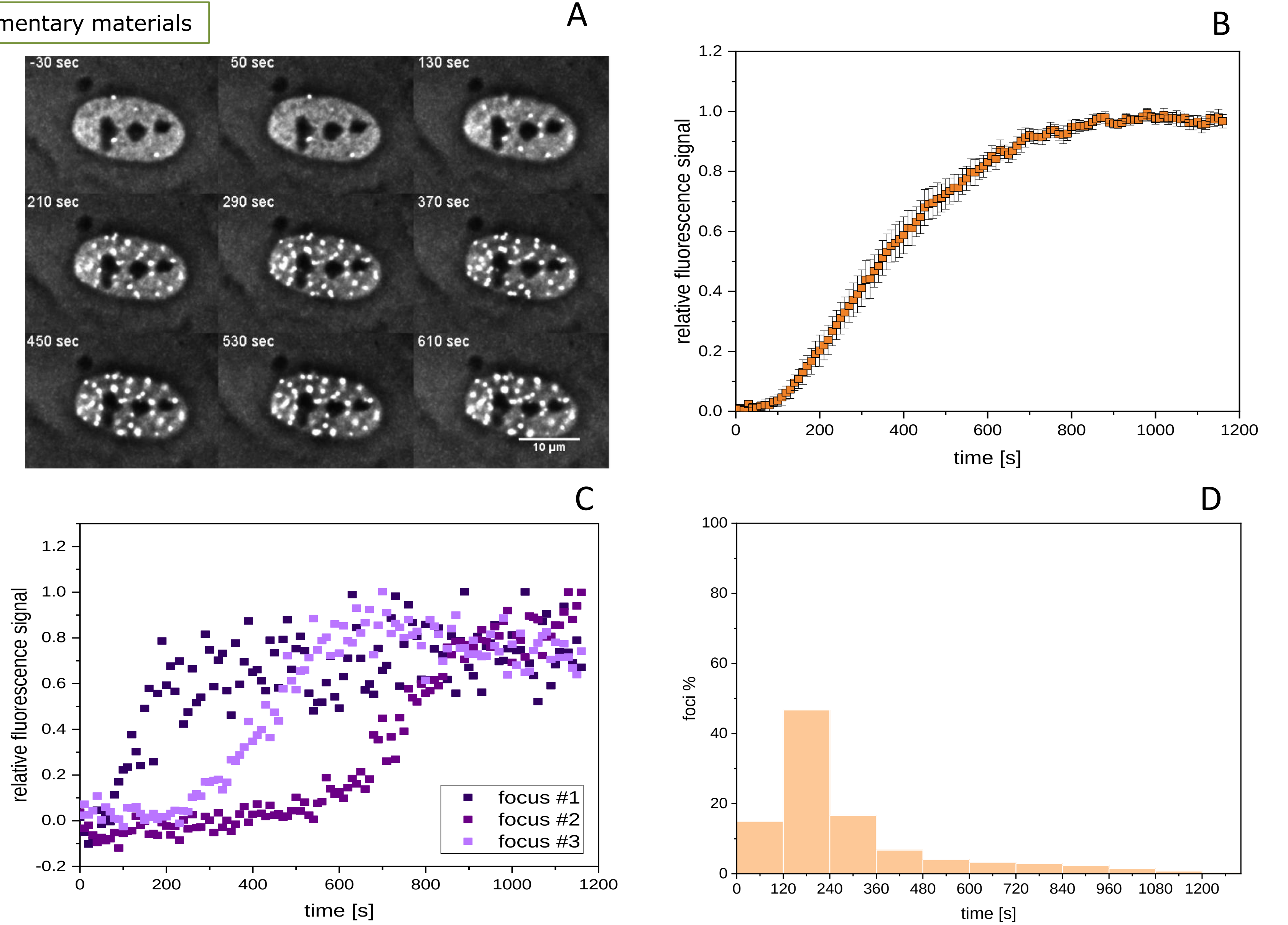

Figure S6: Real time recruitment of 53BP1-GFP in U2OS cells after irradiation with x-rays : (A) Selected images of a movie of a U2OS nucleus expressing 53BP1-GFP showing accumulation at DSBs after irradiation with 0.85 Gy x-rays. (B) Recruitment kinetics of 53BP1 to DNA DSBs induced 35kV x-rays is characterized by a sigmoidal recruitment behavior. (n=20 nuclei, Error bars 95% CI). (C) Selected traces of 53BP1-GFP recruitment to individual off-track foci showing clearly delayed onset of responses at some DSBs. (D) Analysis of lag phases of x-ray induced 53BP1 foci formation showing a broad distribution of delayed onset of 53BP1 recruitment after x-rays (n=888 RIF). Image montage in (A) was done using ImageJ 1.48v (https://imagej.nih.gov). Graphs were generated using Origin Pro V.2019 (Originlab Corp., Northampton, MA, USA).

supplementary materials

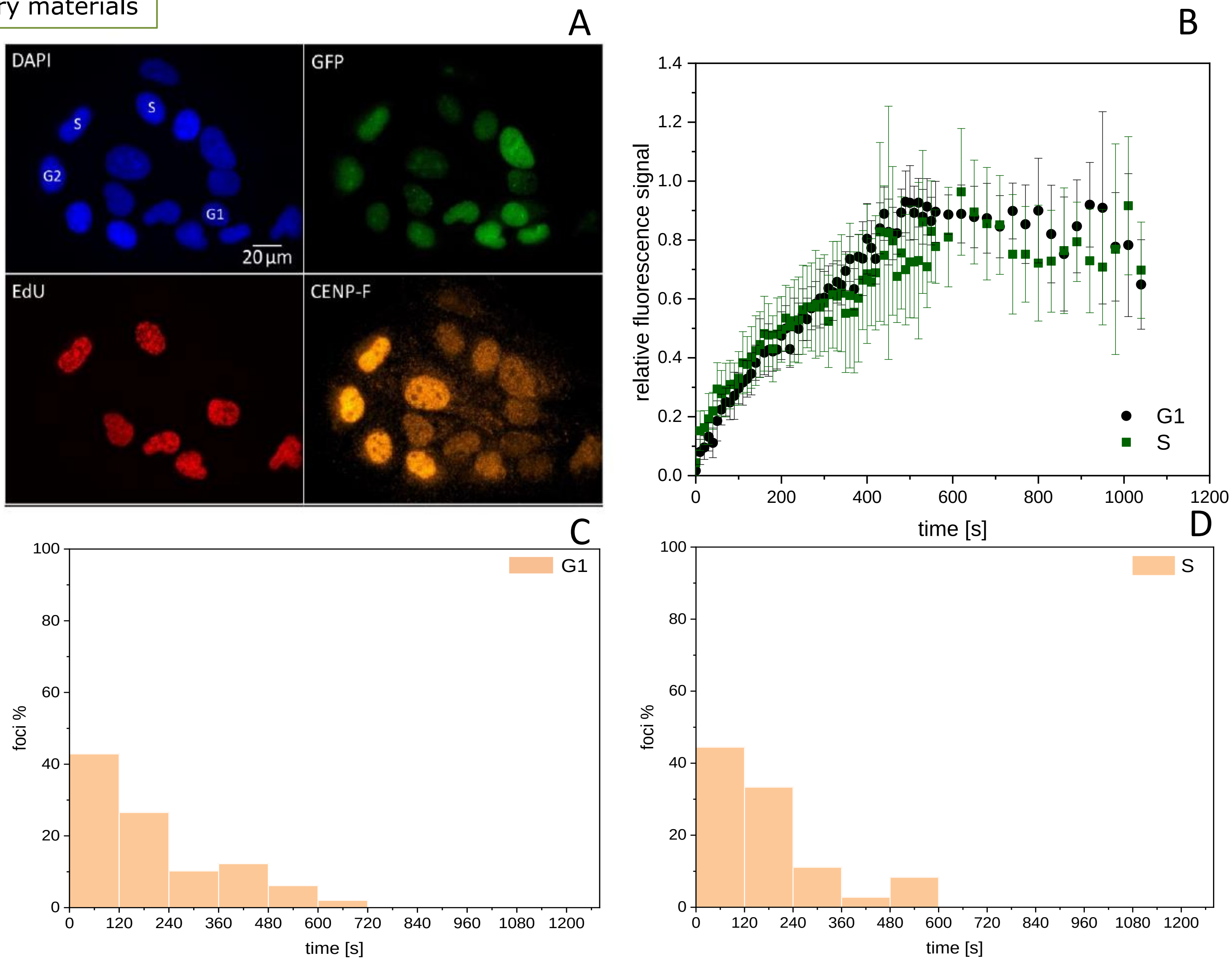

Figure S7. Delayed recruitment is not S-phase dependent (A) Representative images of the cell cycle phases staining in U2OS-Nbs1-GFP cells (G1: EdU negative, CENP-F negative; S: EdU positive, CENP-F negative/positive; G2: EdU negative, CENP-F positive). (B) Recruitment kinetics of Nbs1-GFP factor after 0.7 Gy X-ray irradiation of U2OS cells in G1- (blue) compared to S-phase (green)(G1: n=49 RIF in 6 nuclei, S: n=36 RIF in 5 nuclei; error bar = 95% CI). After live-cell measurement of recruitment kinetics, cells were labelled with an EDU-puls, subsequently fixed and immunostained against the cell-cycle marker CENP-F. After revisiting the cell, cell cycle phase was determined by fluorescence microscopy as in (A). Lag phase distribution of NBS1-GFP foci development of U2OS cells in (C) G1 phase (n=49) and (D) S-phase (n=36). Image montage in (A) was done using ImageJ 1.48v (https://imagej.nih.gov). Graphs were generated using Origin Pro V.2019 (Originlab Corp., Northampton, MA, USA).

supplementary materials

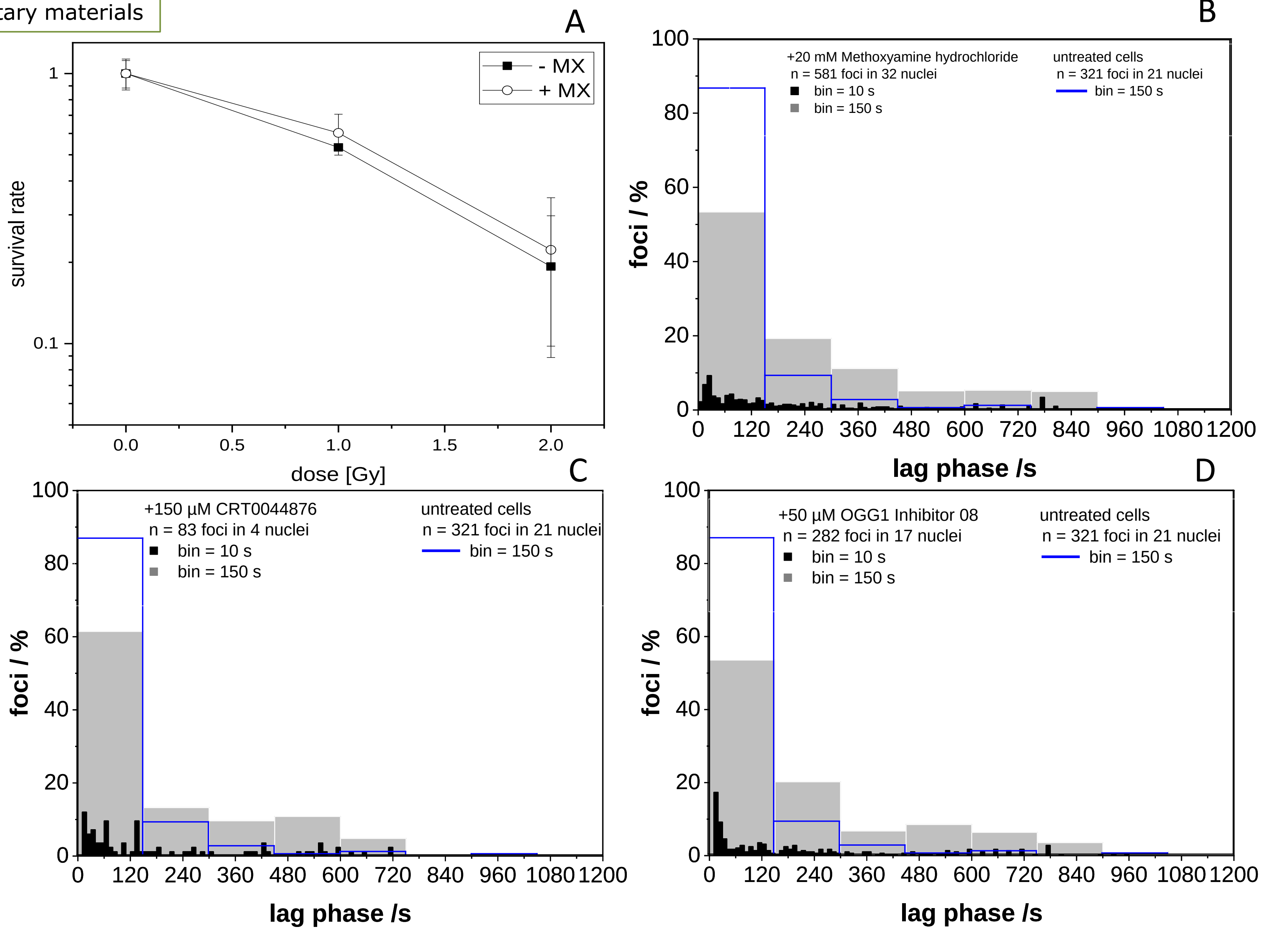

Figure S8. (A) Survival rate of U2OS_NBS1_GFP cells after treatment with x-ray 0-2Gy and t=1 h methoxyamine hydrochloride. Lag phase distribution of NBS1 after treatment U2OS cells with single inhibitors: (B) 20 mM methoxyamine hydrochloride (APE1 inhib.) t= 2 h, (C) 150 µM CRT0044876 (APE1 inhib.) t= 1.5 h, (D) 50 µM OGG1 inhib t= 1 h. Graphs were generated using Origin Pro V.2019 (Originlab Corp., Northampton, MA, USA).

supplementary materials

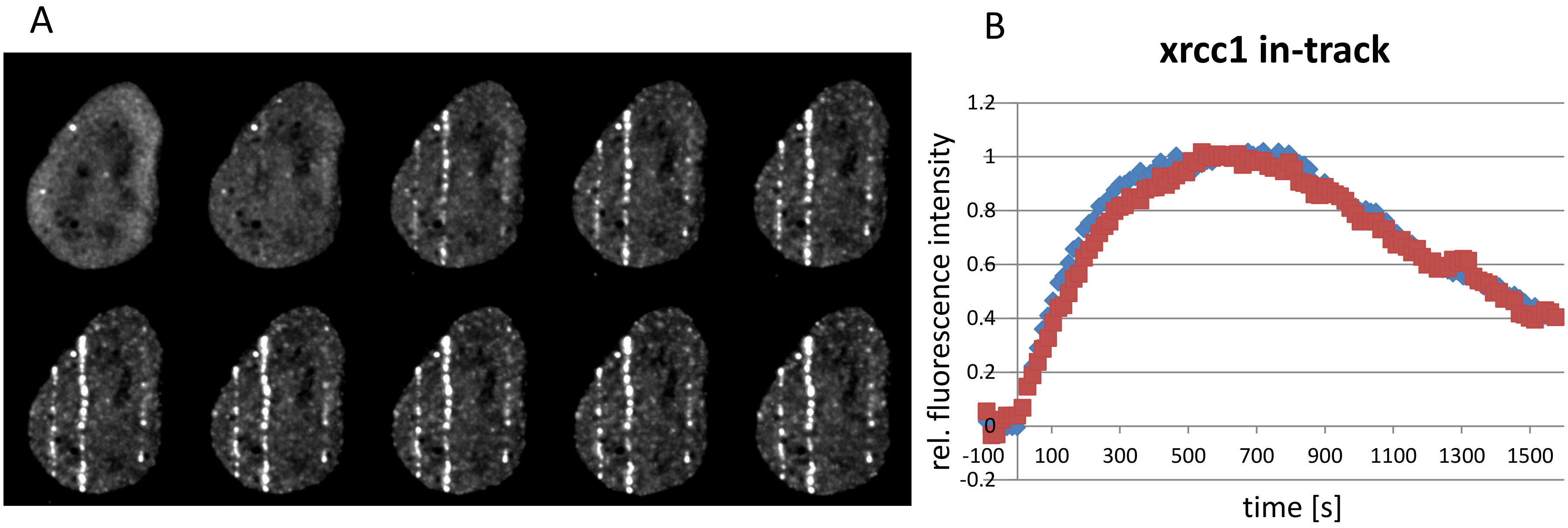

Figure S9: Real time recruitment of EGFP-XRCC1 in HT1080 cells after irradiation with 750 MeV/u U-ions: (A) Selected images of a movie of aHT1080 nucleus expressing EGFP-XRCC1 showing a clear and fast accumulation at the ion trajectories after irradiation. The contrast was enhanced by changing the LUT to emphasis the radiation induced grainy pattern outside the tracks. For original data see Supplemental movies. (B) Recruitment kinetics of XRCC1 to uranium tracks for two different nuclei showing the absence of a pronounced lag-phase as well as the transient binding despite the massive DNA damage induced in the track. Different to the DSB factors NBS1 and 53BP1, the XRCC1 signal intensity peaks around 10 min post-irradiation. Image montage in (A) was done using ImageJ 1.48v (https://imagej.nih.gov). Graph (B) was generated using Microsoft Excel 2010 (Microsoft Corporation, USA).
